# Practical Guide to Large Amplitude Fourier-Transformed Alternating Current Voltammetry—What, How, and Why

**DOI:** 10.1021/acsmeasuresciau.4c00008

**Published:** 2024-05-07

**Authors:** Natalia G. Baranska, Bryn Jones, Mark R. Dowsett, Chris Rhodes, Darrell M. Elton, Jie Zhang, Alan M. Bond, David Gavaghan, Henry O. Lloyd-Laney, Alison Parkin

**Affiliations:** † Department of Chemistry, University of York, Heslington, York YO10 5DD, United Kingdom; ‡ SciMed, Unit B4, The Embankment Business Park, Vale Road, Heaton Mersey, Stockport SK4 3GN, United Kingdom; § Alvatek Ltd.,Unit 11 Westwood Court, Brunel Road, Southampton SO40 3WX, United Kingdom; ∥ School of Engineering and Mathematical Sciences, La Trobe University, Bundoora, Victoria 3086, Australia; ⊥ School of Chemistry and the ARC Centre of Excellence for Electromaterials Science, Monash University, Clayton, Victoria 3800, Australia; # Department of Computer Science, University of Oxford, Wolfson Building, Parks Road, Oxford OX1 3QD, United Kingdom

**Keywords:** electrochemistry, Fourier-transformed voltammetry, large amplitude ac perturbation, fast electron transfer reactions, redox chemistry, solution voltammetry

## Abstract

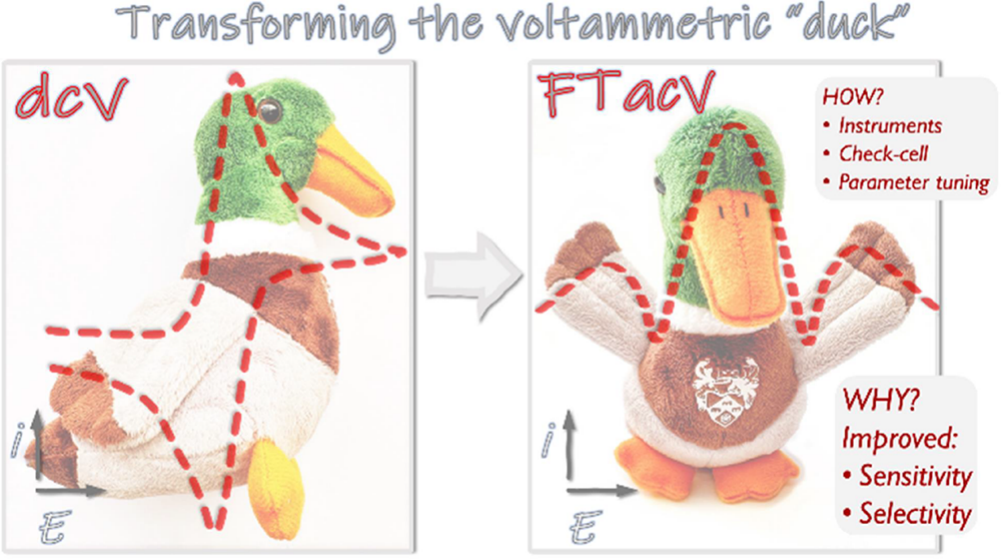

Fourier-transformed alternating current voltammetry (FTacV) is a technique utilizing a combination of a periodic (frequently sinusoidal) oscillation superimposed onto a staircase or linear potential ramp. The advanced utilization of a large amplitude sine wave induces substantial nonlinear current responses. Subsequent filter processing (via Fourier-transformation, band selection, followed by inverse Fourier-transformation) generates a series of harmonics in which rapid electron transfer processes may be separated from non-Faradaic and competing electron transfer processes with slower kinetics. Thus, FTacV enables the isolation of current associated with redox processes under experimental conditions that would not generate meaningful data using direct current voltammetry (dcV). In this study, the enhanced experimental sensitivity and selectivity of FTacV versus dcV are illustrated in measurements that (i) separate the Faradaic current from background current contributions, (ii) use a low (5 μM) concentration of analyte (exemplified with ferrocene), and (iii) enable discrimination of the reversible [Ru(NH_3_)_6_]^3+/2+^ electron-transfer process from the irreversible reduction of oxygen under a standard atmosphere, negating the requirement for inert gas conditions. The simple, homebuilt check-cell described ensures that modern instruments can be checked for their ability to perform valid FTacV experiments. Detailed analysis methods and open-source data sets that accompany this work are intended to facilitate other researchers in the integration of FTacV into their everyday electrochemical methodological toolkit.

## Introduction—The What of FTaCV

Voltammetry employing cyclic potential–time sweeps (herein referred to as “direct current” voltammetry, or dcV) is a widely used, simple electrochemical technique. In the present era, the traditionally employed analogue linear potential waveform has been substituted by a digitally generated staircase. In the most fundamental application, dcV has great power in enabling synthetic chemists to “fingerprint” the redox behavior of their compounds.^1^ In a more sophisticated and complex analysis, mathematical models of the electron transfer reaction can be utilized to generate simulations of the experimental data and optimization algorithms can be employed to find the numerical model parameters that provide a “best fit” to the experiment.^2^ This paper aims to showcase how the incorporation of large amplitude Fourier-transformed alternating current voltammetry (FTacV) into the experimental voltammetric toolkit can produce a data set comprising a series of harmonics that can be visually analyzed in a simple manner to provide a fingerprint for the redox activity of electroactive moieties under conditions where dcV analysis is challenging or exhibits insufficient sensitivity. This increased sensitivity is obtained by superimposing a periodic signal onto a dcV ramp and filtering the resultant total FTacV current in the frequency domain, as obtained by Fourier-transformation.

Initial method developments focused on small amplitude (typically 5 mV or less) periodic perturbations, resulting in a detailed quantitative analysis of the fundamental (first) and second harmonic components.^3−5^ After decades of method evolution, which has shifted onto the use of large amplitudes (50–250 mV) allowing access to higher order harmonics,^6−24^ the FTacV technique can now be performed using commercially available electrochemical workstations.^25^ Thus, a wider community of experimentalists who do not need to be experts in building instrumentation or software development can access this technique. In the study herein, we describe what chemical insight can be gained from conducting a FTacV experiment, detail how to ensure that a commercial instrument can carry out the measurements with sufficient accuracy and data resolution, and illustrate why it can be advantageous to conduct voltammetry experiments using large amplitude FTacV instead of the more traditional dcV technique.

As has been previously described clearly and comprehensively by Elgrishi et al. in their introductory guide to cyclic voltammetry,^1^ electroanalytical experiments are predominantly conducted using a three-electrode setup within a so-called electrochemical “cell”.^1^ Voltammetry requires the input of a potential–time perturbation at the working electrode; the potential is set versus the reference electrode. The experimental output is a measure of the resultant current flow between the working electrode and the counter (sometimes referred to as auxiliary) electrode.^26^ An electrochemical workstation, often marketed as a “potentiostat”, is used to control and monitor the experiment.^9^ Historically, the electronic definition of “potentiostat” is simply a voltage source that is able to vary its output potential in response to changes in the resistance across a circuit, i.e., it is the circuit required to control/set (“stat” stems from the Greek word “statos”, defined as standing or set) the potential at the working electrode.^9,27^ However, in modern electrochemical terminology, “potentiostat” is frequently used as a broader catch-all term to describe an entire analytical instrument containing many internal circuits designed to not only control the potential of the working electrode but also measure currents. Indeed, most so-called “potentiostat” instruments can be used as galvanostats, i.e., they can be used to control the current rather than the potential. In keeping with the modern chemistry nomenclature, we herein utilize the term “commercial potentiostat” to describe an instrument capable of both applying a controlled potential and measuring the resultant current.^1^

The collected data from a dcV experiment is presented as a so-called “voltammogram” that displays the current output (*y*-axis) as a function of the input potential (*x*-axis), with the IUPAC convention defining the oxidative current as positive and reductive current as negative.^1,28^ The total current is a sum of both the “Faradaic” and “non-Faradaic” current contributions. “Faradaic” refers to the current generated by formal electron transfer between electroactive species and the working electrode, while the term “non-Faradaic” current defines the electron flow due to the rearrangement of charged species, including electrolyte ions, at the electrode–solution interface.^26^ Thus, in a similar manner to spectroscopy experiments where solvent and cuvette absorbance features must be considered, it is logical that electrochemical control experiments are performed in the absence of the analyte to assess the extent of “background” contributions within the experiment.^1^ However, it is worth noting that the background current may be modified by the addition of an electroactive species so a background subtraction of the two data sets is inaccurate and hence not advised. Provided that a sufficiently “blank” analyte-free measurement has been obtained, a simple visual interpretation of a dcV voltammogram can be performed. From visual analysis, one can determine the potential window over which a compound displays electroactivity (based on the *x*-axis position of the Faradaic current response), and the extent of chemical reversibility for the electrochemical processes under investigation (based on the ratio of the oxidative to reductive Faradaic current). When experiments are conducted using sufficiently low scan rates, *v*, i.e., small potential–time gradients, the oxidized (Ox) and reduced (Red) species are assumed to be under equilibrium conditions (eq 1) at each applied potential and so the Nernst eq (eq 2) can be used to interpret the Faradaic current contributions; *E* refers to the potential of the working electrode with respect to a reference electrode, *E*^0^ is the standard potential of the redox process versus the same reference electrode, *E*^0’^ is the formal potential taking into account the activity coefficients of both Red and Ox, *x* is the distance from the electrode surface, *R* is the universal gas constant, *T* is the temperature, *n* is the number of electrons, *F* is Faraday’s constant, and *Q* is defined as the reaction quotient.
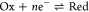
1
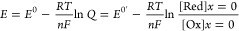
2

Classically, dcV experiments aim to investigate the redox behavior of molecular species dissolved in solution. To obtain easily interpretable data (i.e., the classic “duck”-shaped response of the transient voltammetry for a reversible electron-transfer process), experimentalists should select (i) a sufficiently high analyte concentration to ensure the Faradaic current dominates over the non-Faradaic contributions, (ii) solvent, electrolyte, and gas atmosphere with no redox activity observable within the potential window of interest, and (iii) a working electrode that is both inherently nonredox active under the experimental conditions and has minimal reactivity with the species under investigation. Achieving such conditions can be practically demanding. Furthermore, the production of large amounts of novel compounds can be challenging; therefore, electrochemistry experiments that require the analyte in either large volumes, high concentrations, or both may not be feasible. The potential window of activity for some electroactive compounds overlaps with the reduction of oxygen, requiring removal of the latter by employing an inert gas such as argon or nitrogen, or solvent activity (e.g., proton reduction) may overlap with the process of interest. In this paper, we aim to illustrate that instead of optimizing the experimental setup to support dcV measurements (which may involve acquiring bespoke, low-volume electrochemical cells, setting up anaerobic gas environments, or embarking on a wide solvent and/or working electrode screening process) experimentalists may find it is more beneficial to optimize their electrochemical methodology instead.

**Figure 1:**
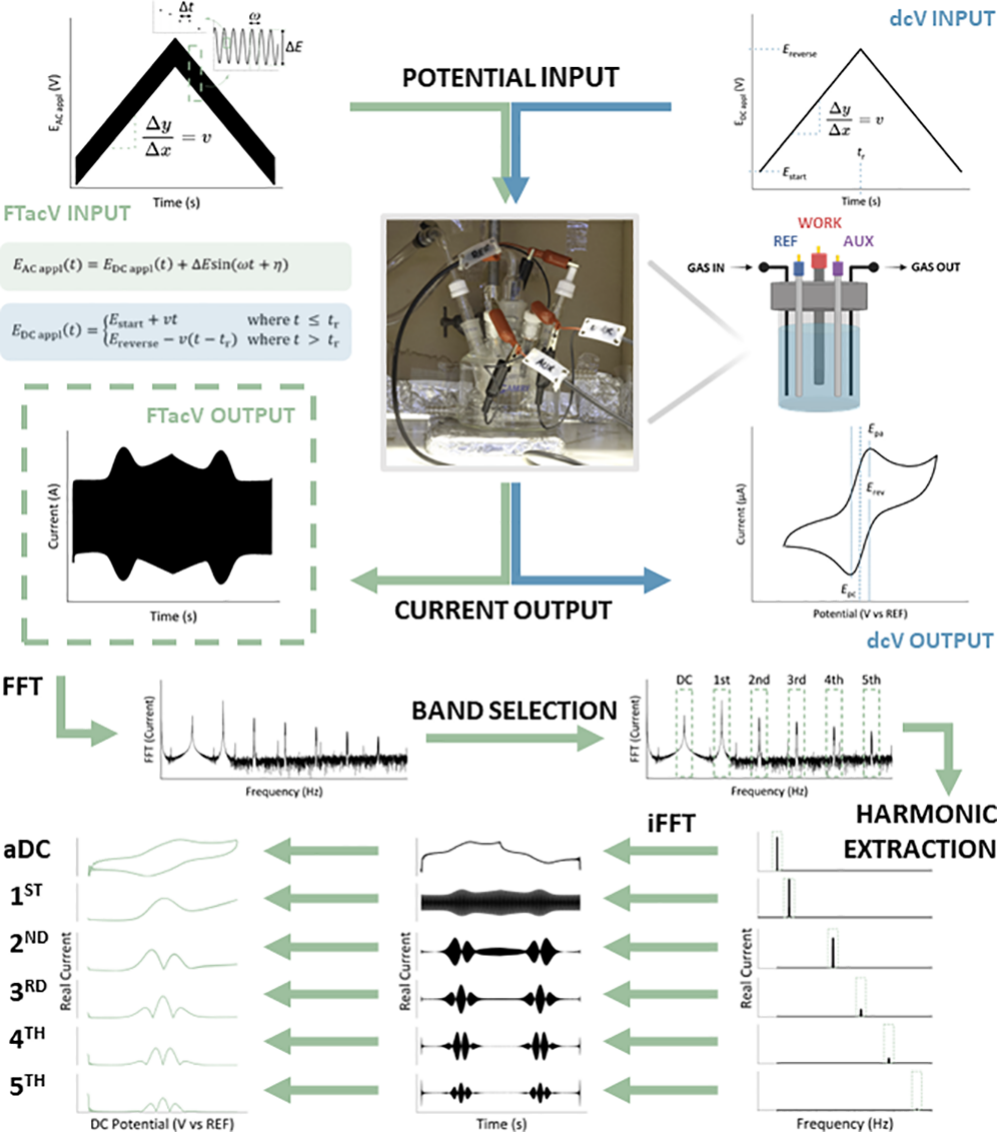
Schematic illustrating the process of collecting and extracting data for a FTacV experiment. The top row contrasts the FTacV potential input (left) with the dcV potential–time input (right) and provides graphical descriptions of the key experimental input parameters required to generate both potential inputs; the mathematical definitions are shown in green and blue boxes for FTacV (eq 4) and dcV (eq 3), respectively. The subsequent row shows the photograph of the electrochemical cell (center, picture taken by Dr S. Akkad for use in this figure) used to collect all the solution voltammetry data and the corresponding schematic diagrams of the setup (right). In the panel below, the typical current outputs for FTacV (left) and dcV (right) for a reversible redox-active analyte are shown. The following two bottom panels graphically explain the process of obtaining FTacV harmonics from the current output. The total current is Fourier-transformed (FTT) to obtain a spectrum containing the current at each frequency. Individual current signals (harmonics) are identified using band selection of the Fourier spectrum; harmonic peaks are found at integer multiples of the input frequency, *n*ω. The spectra are filtered as detailed in Box 1 and inverse Fourier-transformed (iFFT) to obtain the individual harmonics. This can either generate total current harmonic or envelope plots, with the latter being more conventional, which can be plotted versus time or DC potential; the conversion between the two *x*-axis is detailed in Box 2. Data was collected on the “Home-Build” potentiostat using 0.10 mM Fc in MeCN containing 0.25 M [NBu_4_][PF_6_] as the supporting electrolyte.

As noted above, Smith and his colleagues extensively explored small amplitude FTacV, where typically Δ*E* did not exceed 5 mV. Over the past 20 years, we have been developing the large amplitude version of the FTacV method, employing typically a Δ*E* of 80 mV, allowing us to generate 10 or more higher order harmonics for reversible electron transfer processes. Within this paper, we demonstrate how and why nonelectrochemical specialists can and should incorporate this large amplitude FTacV methodology within their standard electrochemical characterization toolkit. In standard cyclic voltammetry measurements, the DC potential input, *E*_DC appl_(*t*), as defined in eq 3, is applied;
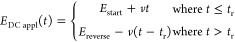
3where *v* is the scan rate, *t* is the time, *E*_start_ is the starting potential of the first sweep, *E*_reverse_ is the potential at which the sweep is reserved and returns to the *E*_start_, and *t*_r_ is the time at which the reversal occurs, defined as ((*E*_reverse_ – *E*_start_)/*v*) (Figure 1). As noted above, modern instruments supported by a digital computer do not generate a true linear potential–time ramp; instead, this is approximated via a staircase. The FTacV method described herein simply adds a sinusoidal wave perturbation to the DC potential, although other kinds of periodic waveforms have been explored in the literature.^29,30^ For a single sine wave superimposed onto a DC ramp, *E*_AC appl_(*t*) (eq 4) is given by
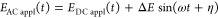
4where *E*_DC appl_(*t*) is as defined above, Δ*E* is the amplitude of the sinusoid, ω is the frequency of the applied sine wave, and η is the phase (Figure 1). As with the “linear” DC portion of the experiment, in the modern digital area, a sine wave is only an approximation of a series of small potential steps.

In FTacV, as described herein, a sufficiently large Δ*E* input value must be chosen to induce a substantial nonlinear current response, alongside a constant sampling time, Δ*t*, which is required for algorithmic Fourier-transformations.^8,12,13,16,19,31,32^ Provided that these measurement conditions are met, after collecting the FTacV data, the resulting current–time output can undergo fast Fourier-transform (FFT) processing to generate an absolute Fourier power spectrum, which comprises current contributions at integer-multiples of the input sine frequency, ω (Figure 1). The absolute Fourier-transform is defined as the square root of the sum of the squares of the real and imaginary components of the Fourier spectrum. The Fourier spectrum generated by numerical FFT algorithms in most programming contains responses at both positive and negative frequencies; while the latter is clearly nonphysical, it is a consequence of the complex exponential representation of sinusoids used in Fourier analysis. For a real (noncomplex) signal, the response at these “negative frequencies” is identical to the corresponding positive frequency response and thus contains no additional information. Selection and subsequent inverse fast Fourier-transform (iFFT) of the individual current responses are performed to produce distinct current–time outputs, each referred to as the “*n*th harmonic”, where *n* refers to the scalar factor applied to the input frequency. It is often more convenient to view the harmonic components as current envelope plots, rather than visualize the oscillation in the current, as illustrated in Figure 1.

Fourier processing of the data forms a crucial part of FTacV as both the FFT and iFFT methods are utilized as a filtering step that facilitates the separation of current contributions on the basis of kinetics. Band selection and the subsequent iFFT of the current–time output within the box filter centered at 0 Hz produces the so-called “aperiodic DC component” that comprises the current flowing in response to the DC component of the potential–time oscillation. We therefore usually graphically represent the aperiodic DC component in a plot of current versus the DC potential, rather than a current versus time plot. The methodology for converting between time and DC potential is detailed in Box 2. However, it should be noted that the aperiodic DC component is not identical to a conventional voltammogram from an dcV experiment as amplitude-dependent Faradaic rectification distorts the response. Analogous processing of the higher harmonics, i.e., current contributions at frequencies equivalent to integer multiples of the input frequency, results in current–time outputs dominated by Faradaic current arising from fast electron transfer processes, which can also be plotted against DC potential. A range of windowing processes can also be applied at this stage, and we have found this to be of substantive value during data simulation. However, we note that such operations distort the data (Figure S4) and therefore have not been used throughout the paper. Access to the higher order, for example, fourth to sixth harmonics, allows for visual interpretation of the data even when the non-Faradaic current, competing slow electron transfer, or otherwise irreversible processes overwhelm the signal of interest in the aperiodic DC component, as such processes are not observable beyond the third harmonic. Herein, we showcase this through the investigation of the reversible electron transfers of Fc^0/+^ (eq 5) and [Ru(NH_3_)_6_]^3+/2+^ (eq 6) redox couples, standard calibrants in nonaqueous and aqueous electrochemical experiments, respectively.
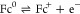
5
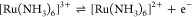
6

Throughout the method development process of large amplitude FTacV and complementary techniques,^7,8,11−14,16,18,19,22−24,32,33^ we have always employed bespoke, homebuilt instruments (herein referred to as the “Home-Build”) containing 18-bit analogue-to-digital and digital-to-analogue converters that facilitate the production of a reliable and stable measurement, which results in a large experimental dataset of 2^15^ bytes.^7,9^ However, since this method development work started over 20 years ago, significant advances in electrochemical impedance spectroscopy (EIS) and commercial potentiostat hardware have been made. As a result, most modern electrochemical instruments are capable of waveform generation utilizing high frequency sine waves. Within this practical guide, we describe a “check-cell” design, comprising only simple electrical components, which allows facile investigation of the accuracy with which an instrument can perform FTacV experiments. We highlight the necessity for high sampling frequency and provide sample data to demonstrate the importance of low resistance within the electrochemical cell setup. For the purpose of pedagogical clarity, the experimental data within this paper solely focus on a simple, one-step reversible elementary electrode reaction on an inert working electrode, involving species dissolved in solution. However, arguably, a more attractive use of FTacV is for the study of more complex analytes, such as but not limited to, surface-confined or immobilized species, multistep electrode reaction, or multistep reactions with adsorption–desorption steps.^23,25,34−37^

Box 1 **How to extract harmonics?**

Deconvolution of the FTacV current into individual harmonics requires a number of steps. This can be achieved using a “top hat” filter analysis:

- Process the total current using the fast Fourier transform (FTT) algorithm; this is inbuilt in almost all numerical programming languages, e.g., MATLAB.
- For each harmonic to be plotted, create a box centered on the desired harmonic peak, where the width of this box is a fraction of the input frequency; this fraction should be determined through the inspection of the Fourier spectrum. Throughout this paper, we have used a width of 0.1ω, where ω is the input frequency.
- Make a copy of the Fourier spectrum for each harmonic and zero out all of the spectrum except the values within the box.
- The resulting filtered spectrum is then processed through an iFFT algorithm producing a complex signal. The real, imaginary, and absolute (defined as the square root of the sum of squared real and imaginary components) values of the harmonics can be plotted.
- To simplify the presentation of the harmonics, an envelope plot (which traces the maximum values of the current) is frequently chosen.

Box 2 **How to perform time-to-potential conversion?**

The universal standard is to plot voltammetry data as current on the *y*-axis versus the applied potential on the *x*-axis, to facilitate reading off the position of the peak versus potential. This is also the case for FTacV. However, plotting individual time-domain harmonics against the AC potential can result in a graph which is visually difficult to interpret. Therefore, it is more convenient to plot harmonics versus the DC component of the total input.

The most straightforward way to obtain the DC potential values is to apply the parameters from eq 3 for the generation of the waveform and input them into eq 2 to mathematically generate the DC potential.

In the resulting plot of harmonic current versus DC potential, the midpoint of each harmonic (for even harmonics, the central trough, and for odd harmonics, the central peak) corresponds to *E*_rev_.

## Materials and Methods

The electrochemical experiments were conducted using three different electrochemical workstations, throughout the text referred to as “potentiostats”. The “Home-Build” potentiostat refers to a bespoke, computer controlled electrochemical workstation, with further references to its manufacture available in the main text, operated using a bespoke “pot” software. The “Ivium” potentiostat refers to a pocketSTAT2 instrument manufactured by Ivium Technologies and supplied by Alvatek Ltd., operated using the associated IviumSoft Software for Windows (updated to the latest version at the time of writing) through the “ChronoAmperometry” method and bespoke profile waveform. The “Gamry” potentiostat refers to a Reference 620 instrument manufactured by Gamry instruments and supplied by SciMed Ltd., operated using a bespoke Python code. All the current–time output data was exported as text files containing three data columns (time, potential, current) which were subsequently processed as described.

All non check-cell experiments were performed using a conventional three-electrode setup comprising a stationary glassy carbon (GC) working electrode (BASi) (Ø 3.0 mm), a platinum wire (Ø 0.2 mm) counter electrode (Electronics Workshop, Department of Chemistry, University of York) and a reference electrode. A platinum wire (Ø 0.2 mm) pseudo reference electrode (Electronics Workshop, Department of Chemistry, University of York) was used for ferrocene (Fc) experiments (conducted in an acetonitrile solvent) and a Ag/AgCl (aqueous 3 M NaCl) reference electrode (BASi) situated within a Luggin capillary was used for [Ru(NH_3_)_6_]^3+^ (in the form of hexaammineruthenium(III) chloride) experiments (conducted in aqueous solution). Before each experiment, the working electrode was polished mechanically on white felt polishing pads (Buehler) using aluminum oxide powder suspensions (MetPrep) of decreasing particle size, 1 (α) and 0.05 (γ) μm, for approximately 1 min per particle size. The electrode was sonicated in water obtained from a Milli-Q purification system for 1 min and ethanol for 1 min to remove any adhered aluminum particles before being dried under a stream of N_2_.

In all plotted voltammograms, the reversible potential of the Fc^0/+^ process is set at zero, while the potential of the [Ru(NH_3_)_6_]^3+^ process is reported versus a Ag/AgCl (3 M NaCl) reference electrode. The conversion factor (c.f.) between the Ag/AgCl reference electrode and the normal hydrogen electrode (NHE) was determined experimentally. The midpoint potential (*E*_1/2_) value, calculated as the average of the reduction (*E*_p_^red^) and oxidation (*E*_p_^ox^) peak potentials for the [Fe(CN)_6_]^3-/4-^ process, where [M] = 10 mM, was measured using cyclic voltammetry in 100 mM phosphate buffer (*I* = 0.464 M, NaCl), pH 7.0. at a GC working electrode. The experimental midpoint value, *E*_m_ (= *E*_1/2_ or half-wave potential), 0.235 V vs Ag/AgCl 3 M NaCl was compared to the literature of 0.425 V vs NHE, to produce a conversion factor of 0.190 V.

All solution experiments were performed in a commercially available electrochemical cell; a Gamry Jacketed EuroCell Kit (SKU 990–00203). Unless otherwise stated, the electrochemical cell was maintained under a positive pressure of compressed argon gas, controlled using a bubbler bottle filled with the experiment solvent (either acetonitrile or water). The exhaust gas was passed through a secondary bubbler bottle filled with water. The electrolyte solution was degassed with argon for at least 10 min prior to commencing an experiment. All experiments were performed on a benchtop setup at ambient temperature which fluctuated between 18 and 22 °C.

All chemical reagents and solvents were used as supplied by the manufacturer; ferrocene (Sigma-Aldrich), hexaammineruthenium(III) chloride (ThermoScientific), tetrabutylammonium hexafluorophosphate (Sigma-Aldrich), potassium chloride (Acros Organics), acetonitrile (HPLC grade, Fisher).

## Results and Discussion

### The How of FTacV: Conducting a Valid Experiment

Consideration of several factors is required to ensure a valid voltammetry experiment, including the instrument hardware, computer software interface, and electrochemical cell components. For FTacV, an experimentalist must be particularly confident that the instrument utilized is able to generate the necessary contiguous potential–time input (as defined in eq 4) and record the resultant current–time output with sufficient accuracy. The analogue potentiostat should possess a fast rise time (microsecond regime) and a good compliance voltage of ≥10 V. The operational performance of an instrument can be explored using a simple to manufacture and operate “check-cell” comprising a resistor in series with either an ideal or a nonideal capacitor (an RC_ideal_ circuit or RC_nonideal_ circuit, respectively).^12^ We provide instructions in the SI for the construction of an example check-cell, including circuit diagrams and photographs (Figure S1), as well as the resistor and capacitance values of each component.

**Figure 2:**
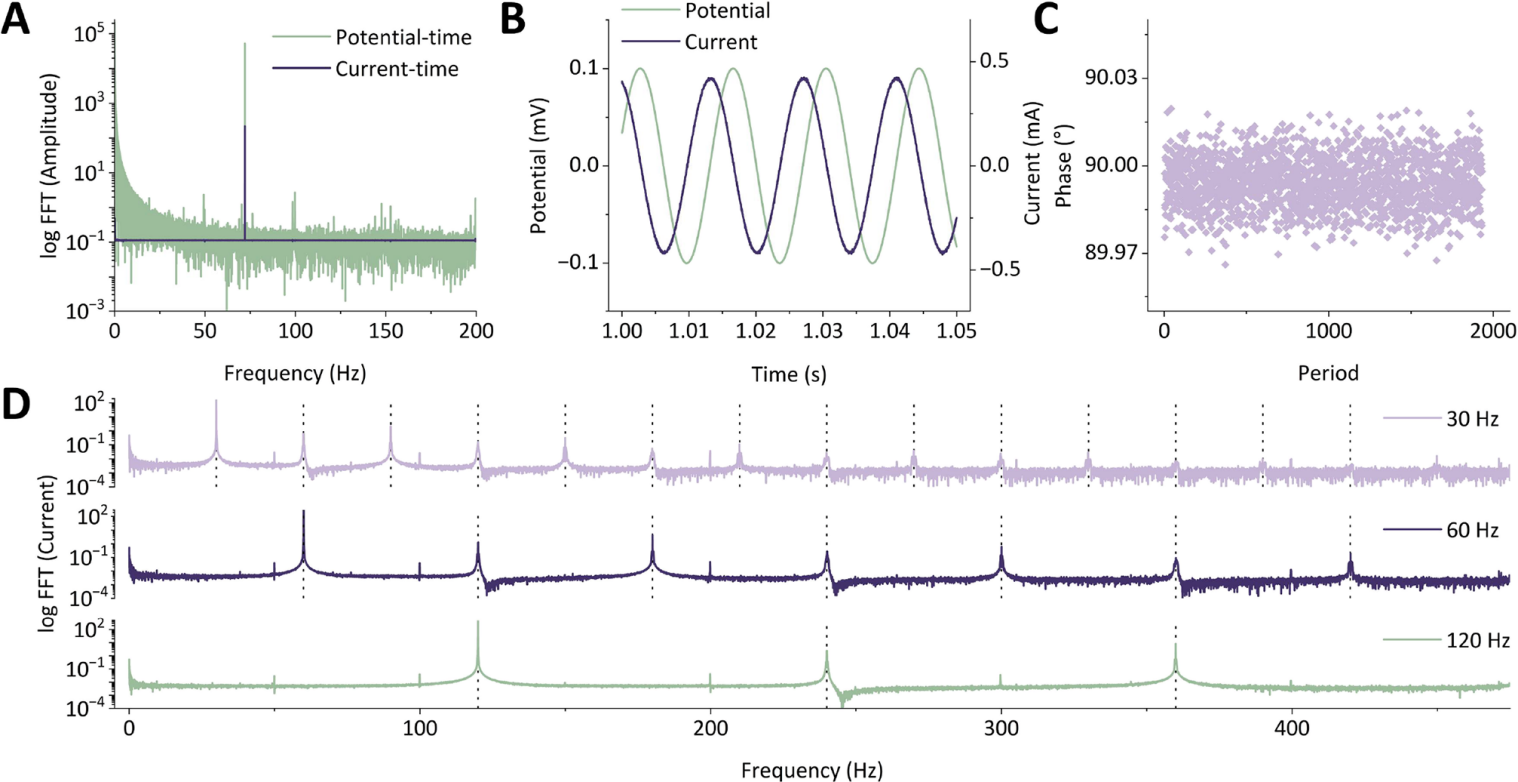
Analysis of the data obtained by conducting cyclic FTacV measurements on the check-cell using the “Home-Build” instrument; ω = 72 Hz, Δ*E* = 100 mV, *v* = 74.51 mV s^–1^, DC potential range = −0.5–0.5 V. (A) Absolute Fourier spectrum generated from the Fourier-transformation of potential–time (green) and current–time (purple) data obtained from the RC_ideal_ check-cell circuit. (B) AC-only components of the potential (green, left-hand *y*-axis) and current (purple, right-hand *y*-axis) data presented in (A), plotted in the time domain. (C) Phase-shift of the current–time sinusoid versus the potential–time sinusoid shown in (B). (D) Absolute Fourier spectrum generated from the Fourier-transformation of the current–time data obtained from the RC_nonideal_ check-cell circuit at frequencies of 30 Hz (lilac), 60 Hz (dark purple), and 120 Hz (green); all other FTacV experimental parameters were unchanged.

In the first instance, the instrument should be connected to the check-cell through the RC_ideal_ circuit in a two-electrode configuration, whereby the working electrode connects to one end of the check-cell (e.g., the resistor) and both the reference and counter electrodes are connected simultaneously at the other end of the check-cell (e.g., the capacitor). Figure 2 shows the data obtained during the testing of our “Home-Build” instrument. The data set shown in Figure 2A–C was collected upon the application of a 72 Hz sine wave with a 100 mV amplitude overlaid on a potential–time ramp of 74.51 mV s^–1^ over a range of 1 V. The experimental parameters are chosen because (i) the mains power frequency in the UK is 50 Hz, and thus, the input frequency cannot share a common multiple with 50 to avoid overlapping with the current contributions for mains noise, (ii) the “Home-Build” instrument is controlled by a software that manipulates the linear-staircase scan range to ensure the data set collected has 2^*n*^ data points and comprises an integer number of sine wave oscillations within the time window of the experiment, often resulting in noninteger *v* values; this is not necessarily the case for commercial instruments.

Figure 2A demonstrates that when FTacV measurements are made on the RC_ideal_ circuit of the check-cell using the “Home-Build” instrument, Fourier-transform processing of the current–time (and potential–time) data generates an absolute Fourier spectrum with the only major peak in the spectrum being present at the input frequency, which for the above data is at 72 Hz. The SI describes in detail the scripting used to produce such Fourier spectrum plots.^38^ This data can be further analyzed to produce a current–time oscillation exhibiting a 90 ± 5° phase shift relative to the input potential–time data, as shown in Figure 2B,C, confirming the ideal capacitor behavior of the circuit under investigation. Subsequently, the “Home-Build” instrument was connected to the RC_nonideal_ check-cell circuit in a two-electrode configuration as described above. Figure 2D demonstrates that when the input parameters remain unchanged, the nonlinearity of the nonideal capacitor gives rise to harmonic peaks at the integer multiples of the input frequency. An illustrative representation of the effect is shown in Figure 2D, where the selection of a 120 Hz input frequency results in a spectrum where the first harmonic is equivalent to the second harmonic of a 60 Hz experiment, which in turn is equivalent to the fourth harmonic of a 30 Hz experiment. Obtaining such data allows the user to confirm the ability of the instrument to generate a current that can undergo iFFT processing to produce distinct harmonics. It is possible to tune in or out of different experimental features by varying the input frequency of the experiment; as explained below, the information contained within each harmonic varies depending on its frequency value.

Having confirmed that the FTacV methodology can be accurately applied to a check-cell, the next step to establishing FTacV measurement methods within the laboratory is to assess whether the components of the chosen three-electrode electrochemical cell introduce any artifacts into the output data, for example, because of high resistance.

**Figure 3:**
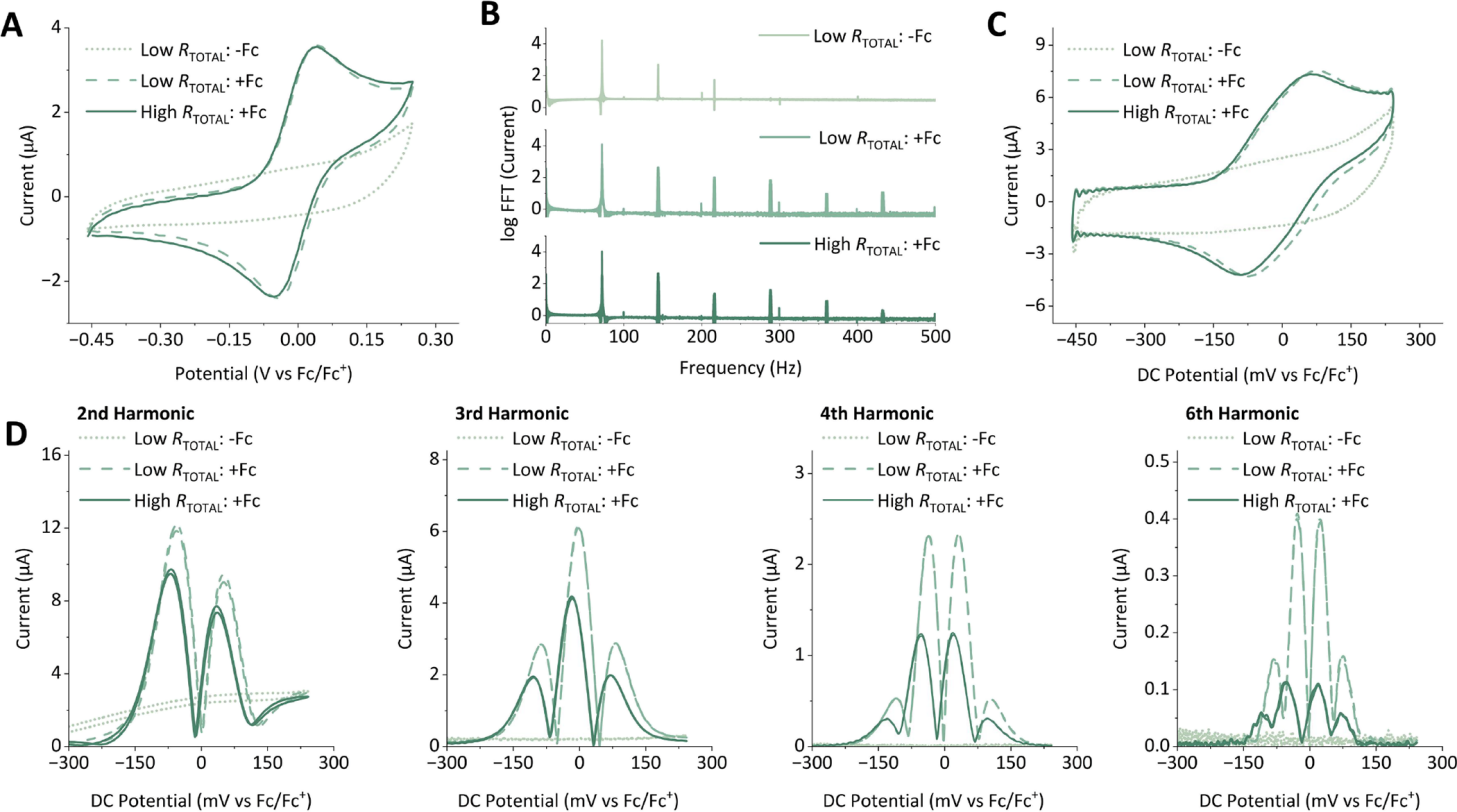
Impact of changes in the total resistance (*R*_total_) on dcV and FTacV experiments for a reversible Fc^0/+^ process in MeCN as a function of the supporting electrolyte concentration, [NBu_4_][PF_6_]. (A) dcV data collected on the “Ivium” potentiostat; *v* = 104.31 mV s^–1^, potential range = −0.45–0.25 V vs Fc/Fc^+^. (B) The Fourier spectrum obtained from the Fourier-transformation of the current–time output data from an FTacV experiment. (C) Aperiodic DC component obtained from iFFT processing of the 0th harmonic from the spectrum shown in (B). (D) Envelope plots of selected harmonic data (2nd, 3rd, 4th, and 6th, as labeled) obtained from iFFT processing of the respective harmonics from the spectrum shown in (B). The FTacV measurements were performed using the “Home-Build” instrument; ω = 72.05 Hz, Δ*E* = 80 mV, *v* = 104.31 mV s^–1^, DC potential range = −0.45–0.25 V vs Fc/Fc^+^. For both dcV and FTacV experiments, the “+Fc” data contained 0.10 mM ferrocene, while “-Fc” indicated ferrocene-free control data; “low *R*_total_” refers to 0.25 M [NBu_4_][PF_6_], and “high *R*_total_” refers to 0.05 M [NBu_4_][PF_6_].

In the typical “duck”-shaped voltammogram response from a conventional dcV solution electrochemistry experiment, a >2.22RT/nF mV peak-to-peak potential separation is the most evident indication of high resistance in a one-electron reversible redox process.^26^ Equally in FTacV, the aperiodic DC component of a cyclic experiment with high resistance resembles a standard “duck”-shaped voltammogram. In high harmonic outputs, the high impedance features are observed as the loss of distinctive splitting patterns.^11^ We illustrate this in Figure 3 by comparing the FTacV data for a reversible Fc^0/+^ process in MeCN collected on the “Home-Build” instrument in the presence of either 0.25 or 0.05 M [NBu_4_][PF_6_] electrolyte salt and the equivalent dcV data measured on an “Ivium” potentiostat. As explained in Bard and Faulkner, the total resistance (*R*_total_) of an experiment can be reduced by increasing the supporting electrolyte concentration, therefore, in Figure 3, we designate “low” *R*_total_ as 0.25 M [NBu_4_][PF_6_] and “high” *R*_total_ as 0.05 M [NBu_4_][PF_6_].^26^ The Faradaic current from the ferrocene process is clearly observable in the sixth harmonic of the “low” *R*_total_ experiment, but the corresponding current is significantly reduced in the equivalent lower electrolyte concentration ferrocene experiment, where *R*_total_ is higher.

In addition to optimization of the electrolyte concentration, minimizing artifacts in FTacV also requires consideration of the reference electrode. Experimentalists may see similar artifacts to those observed in the “high *R*_total_” experiment in Figure 3 if they are using a reference electrode with a frit, such as a commercial Ag/Ag+ reference electrode, as the working-to-reference electrode distance is increased. Thus, throughout the paper, we have employed a Pt wire pseudoreference electrode in all ferrocene experiments, in addition to a glassy carbon working electrode a simple Pt wire counter electron, within a commercial electrochemical cell, as shown in Figure 1.

Comparison of the absolute Fourier spectrum for the ferrocene-free, high electrolyte concentration experiment (“low *R*_total_: -Fc”) in Figure 3B with the Fourier spectrum for the RC_ideal_ check-cell in Figure 2A, obtained using the same instruments, showcases that the non-Faradaic “background” current contributions measured in a real electrochemical experiment are highly nonlinear, i.e., the capacitive current does not resemble the ideal capacitor. However, as is clearly demonstrated in Figure 2D for the RC_nonideal_ check-cell, a careful choice of the input frequency enables selective filtering out of such nonideal capacitive current contributions. Therefore, while dcV solution ferrocene experiments contain nonexcludable non-Faradaic contributions, the higher frequency harmonics of 72 Hz FTacV experiments, measured using the same experimental parameters and the exact same electrochemical cell, report exclusively on the Fc^0/+^ Faradaic process (Figure 3). This can be understood mathematically by considering how the large amplitude sinusoid in eq 4 exposes the nonlinearity of the Faradaic response. The current arising from a redox process is described by an exponential function of e^β(*E*(*t*)-*E*rev)^, where β is a coefficient that varies depending on the model and *E*_rev_ is the reversible potential.^26^ This is the case regardless of whether the Faradaic current is generated by an equilibrium process with reversible kinetics (as modeled by the Nernst equation) or from a nonequilibrium process with quasi- or irreversible kinetics (using models of electrokinetics such as Bulter–Volmer or Marcus–Hush–Chidsey). A function of the form e^*A*sin(*t*)^ can be decomposed to an infinite sum of sines and cosines at frequencies that are integer multiples of *t*. The amplitude of these sines and cosines is a function of the coefficient *A*; in the electrochemical cell, *A* is the amplitude parameter, so by increasing Δ*E*, we make these sinusoids larger, to the point that it is possible to extract them via the Fourier transform.^17,39^

**Figure 4:**
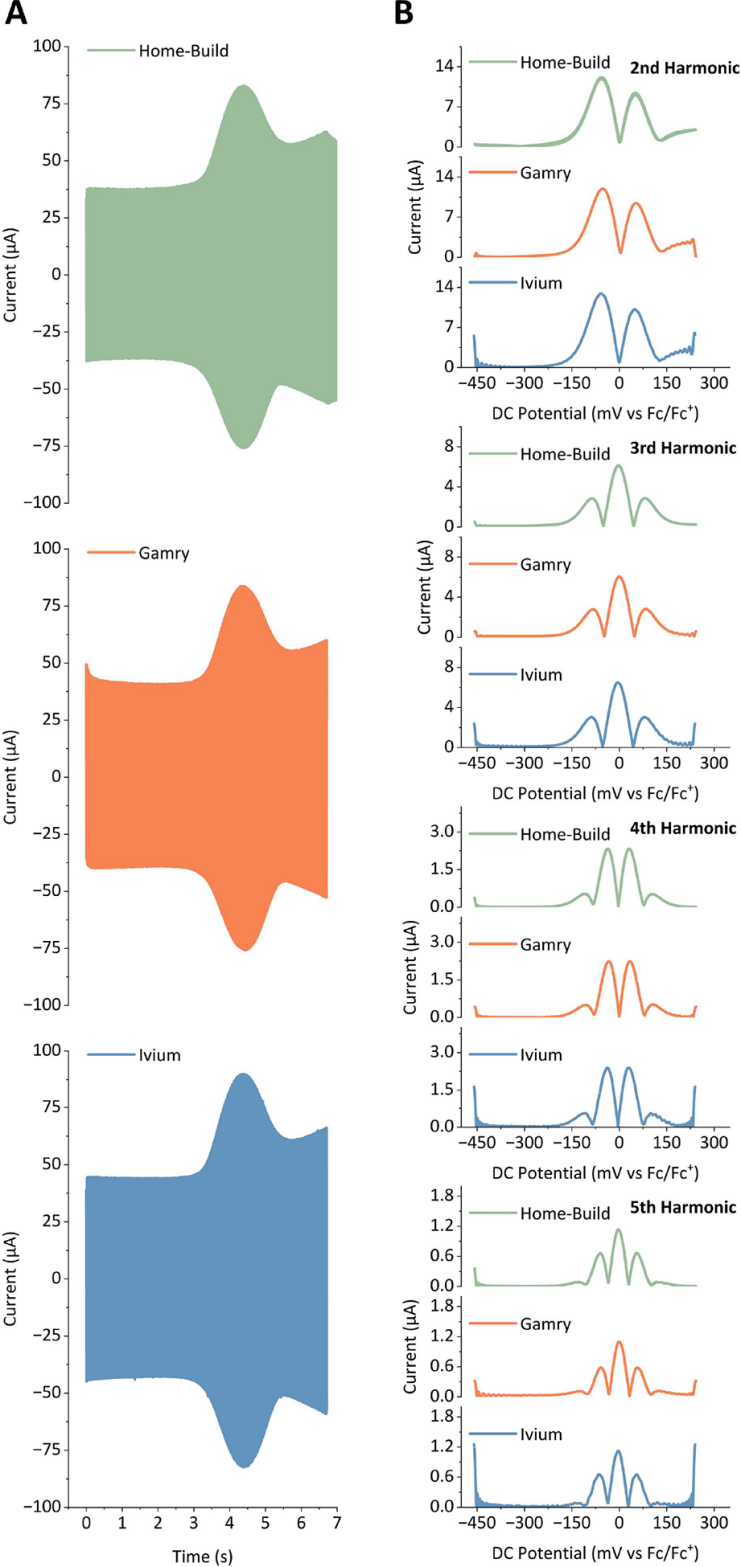
FTacV data for a reversible Fc^0/+^ process in MeCN containing 0.25 M [NBu_4_][PF_6_] as the supporting electrolyte, where [Fc] = 0.10 mM, collected using three different potentiostats: a “Home-Build” instrument (green), a commercial “Gamry” potentiostat (orange), and a commercial “Ivium” potentiostat (blue). (A) Current–time output data for a ferrocene electron-transfer process collected on the individual instruments. (B) Comparison of the 2nd, 3rd, 4th, and 5th harmonic for each instrument obtained from the current–time data in (A) following FFT and iFFT processing. Input parameters were identical for all instruments; ω = 72.05 Hz, Δ*E* = 80 mV, *v* = 104.31 mV s^–1^, DC potential range = −0.45–0.25 V vs Fc/Fc^+^.

Having established the check-cell and simple ferrocene experiments that demonstrate the FTacV method, comparative measurements were performed on the PocketStat2 “Ivium” and Reference 620 “Gamry” potentiostats (Figure 4). The methods for generating the potential–time input files required to run FTacV on these commercial instruments are described in the Materials and Methods alongside the full details of each potentiostat model. For the comparative data sets, we show the data collected using a linear (rather than a cyclic) ramp as the Gamry software is not yet fully optimized for FTacV, so it does not yet have the functionality (upgrades due to arrive in mid-2024) to apply a cyclic DC ramp in combination with a sine wave; instead, Gamry FTacV experiments must employ either a positive or negative potential–time ramp in combination with a sine wave.

The data shown in Figure 4 were collected by simply connecting one potentiostat at a time to the same electrochemical cell containing 0.10 mM ferrocene dissolved in MeCN with 0.25 M [NBu_4_][PF_6_] as the supporting electrolyte. As such, for the same experimental input parameters, we would expect identical responses. This appears to have been achieved for all three instruments as the data shown in both Figure 4A,B is comparable across the three instruments. The most accurate approach to confirm that the output of the experiment is exactly as expected by theory is to compare the current–time output data to a simulation, i.e., compare the experimental data to a computed prediction. However, before predicting a current–time output, it is first necessary to determine the precise nature of the potential–time input, and this is most simply achieved in check-cell confirmation experiments as described herein.

**Figure 5:**
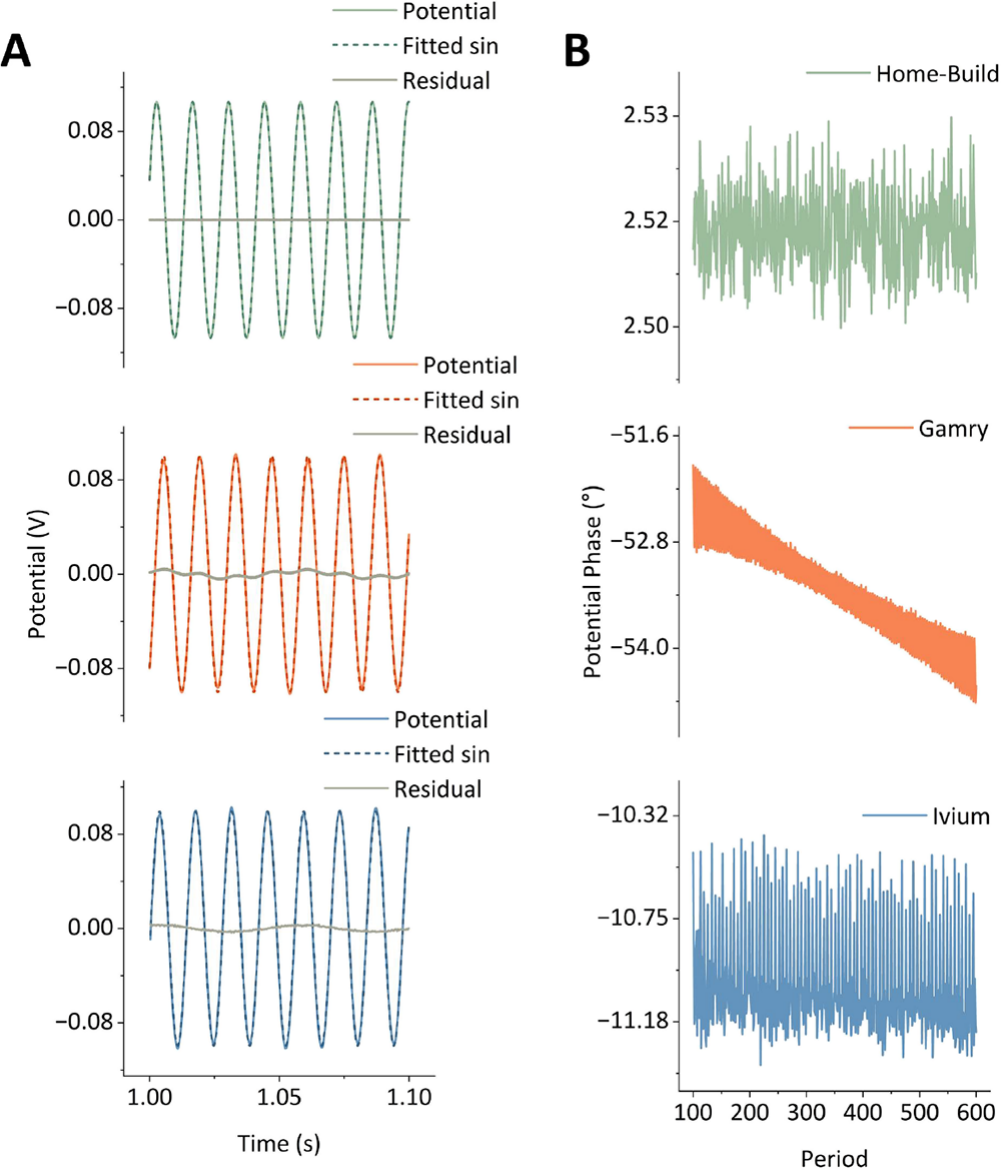
Experimental data using an RC_ideal_ check-cell collected using three different potentiostats: a “Home-Build” instrument (green), a commercial “Gamry” potentiostat (orange), and a commercial “Ivium” potentiostat (blue); ω = 72 Hz, Δ*E* = 100 mV, *v* = 74.51 mV s^–1^, DC potential range = −0.5–0.5 V. (A) Small section of the AC components of the input potential in the time domain (solid coloured line), plotted against a fitted sinusoid (labeled fitted sin) of the form Δ*E*sin(ω*t* + η), where Δ*E*, ω, and η were fitted (dashed coloured line), and the residual obtained from subtracting the fit from the experimental dat (solid gray line). (B) Detected phases of each period of the AC component of an FTacV potential input extracted from the FTacV check-cell experiments.

Figure 5 shows how the RC_ideal_ check-cell can be used to explore the fidelity with which different instruments run an FTacV experiment. We attempt to fit a sinusoid to the AC component of the applied potential (obtained by zeroing out the aperiodic DC component from the potential–time oscillation). As illustrated in Figure 5A, the Home-Build instrument exhibits negligible deviation from an ideal sinusoid, which arises from instrument calibration, which is able to remove any phase inconsistencies.^9^ Conversely, the results show that both commercial potentiostats do not apply a perfect sinusoid—the phase of both varies as a function of time. This can also be observed in the deviation between the applied potential and a pure sinusoid. It should be noted that this does not affect the appearance of the harmonics in the envelope plots, as shown in Figure 4B, where we compare the potentiostats; however, this would be expected to cause challenges when comparing the obtained data to simulations. Furthermore, phasing errors become more apparent and challenging to resolve in regimes of kHz frequencies and above, as well as nA currents and below. In Figure 5B, we analyze the consistency with which the sinusoidal oscillation is achieved throughout the experiment by extracting the phase of each sinusoid in the applied potential–time data set from the three instruments after an FTacV method was run using the same parameters as those defined in Figure 2. Consequently, assessing the phase of the potential–time oscillation generated by a potentiostat is an important check when setting up an instrument to perform FTacV for the first time; we intend to publish a follow-up paper on the simulations of FTacV data generated using our commercial potentiostats.

In most Fourier analysis, the maximum accessible frequency in a Fourier spectrum is the Nyquist frequency, which is determined by the sampling frequency, *f*_s_, as shown by eq 7:
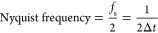
7

**Figure 6:**
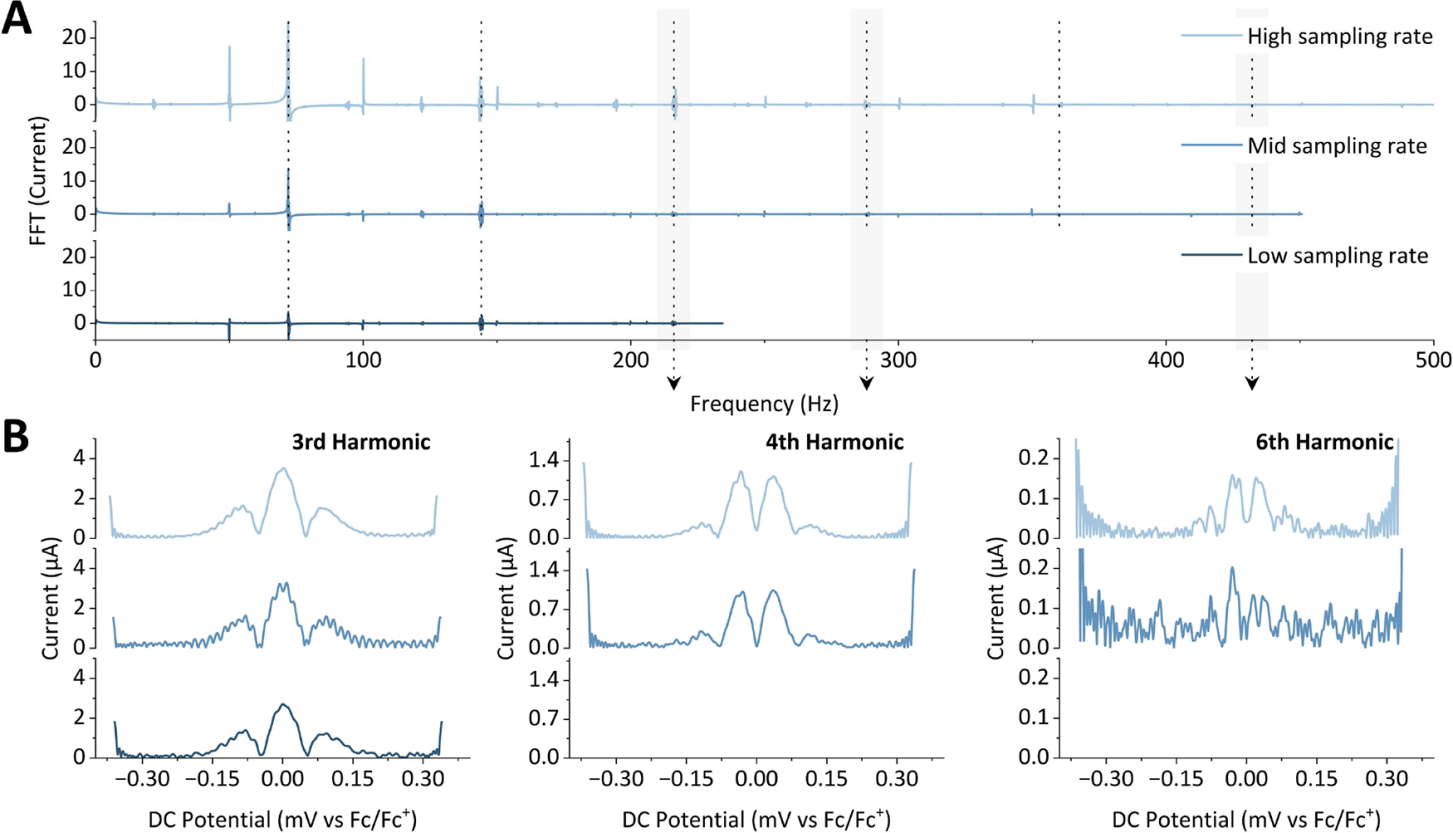
FTacV experiments showcasing the effect of reduced sampling rate performed on a reversible Fc^0/+^ process in MeCN containing 0.25 M [NBu_4_][PF_6_] as the supporting electrolyte, where [Fc] = 0.10 mM, collected using an Ivium potentiostat; ω = 72.05 Hz, Δ*E* = 80 mV, *v* = 104.31 mV s^–1^, DC potential range = −0.45–0.25 V vs Fc/Fc^+^. The time step, Δ*t*, was either 0.00034 s (“high sampling rate”), 0.0011 s (“mid sampling rate”), or 0.0018 s (“low sampling rate”). (A) Fourier spectrum of the three time step conditions, demonstrating the effect of the smaller time step on accessing higher frequencies in the Fourier spectrum. (B) Selected harmonics from each set of conditions, obtained from the iFFT processing of the Fourier spectrum shown in (A).

The effect is illustrated in Figure 6, where the sampling interval time of FTacV data acquired on the Ivium has been purposefully increased from Δ*t* = 0.00034 s (*f*_s_ = 2.9 kHz, “high sampling rate”) to Δ*t* = 0.0011 s (*f*_s_ = 909 Hz, “mid sampling rate”) and Δ*t* = 0.0018 s (*f*_s_ = 556 Hz, “low sampling rate”). Decreasing the sampling rate results in a concomitant decrease in the Nyquist frequency of the Fourier spectrum (i.e., there is a limitation in the maximum accessible harmonic) and a decrease in the signal-to-noise resolution (Figure 6). The signal-to-noise ratio drops because while the total amount of noise in the Fourier-transform of the experiment remains constant regardless of the sampling interval time, at a lower sampling rate, the noise is distributed across fewer frequency bins.^40^

Along with sampling interval time/sampling frequency limitations, all commercial potentiostat instruments also possess a maximum data buffer that limits the total number of data points per experiment. Consideration of this may also limit the choice of FTacV experimental parameters. For a cyclic FTacV experiment, the total time of the experiment, *t*_exp_, is the same as an equivalent dcV experiment, i.e., it is simply given by the time taken to complete the cyclic voltammetry sweep, eq 8:
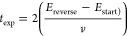
8
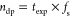
9

The total number of data points, *n*_dp_, is therefore equal to *t*_exp_ multiplied by the sampling frequency, *f*_s_ (eq 9). Thus, although some instruments may have a very high maximum sampling frequency, the Nyquist frequency (and thus the data quality) of an FTacV experiment may still be limited if the data buffer is too small. We illustrate the need to consider these various instrument limitations in Figure S5; for a theoretical experiment measured over a potential window of 1 V, we show the number of points required to access the 30th harmonic as a function of input frequency and scan rate. Such simple considerations demonstrate the utility of splitting one “cyclic” experiment into the two composite “linear” components, as dividing the experiment in two effectively doubles the number of points. Indeed, such an approach is valuable when using the Ivium potentiostat, which in addition to a maximum standard sampling rate of 0.00034 s^–1^, also has a maximum data buffer size of 65000 points for the PocketStat2 as of the time of writing.

We include all our *x*–*y* data from the plots in this paper in a repository. We intend that this combination of highly reproducible experimental setup and freely accessible check-cell data will permit newcomers to the FTacV technique to confidently ensure that their experimental setup and data processing protocols are all working correctly.

### The Why of FTacV: Enhanced Sensitivity and Separability versus dcV

**Figure 7:**
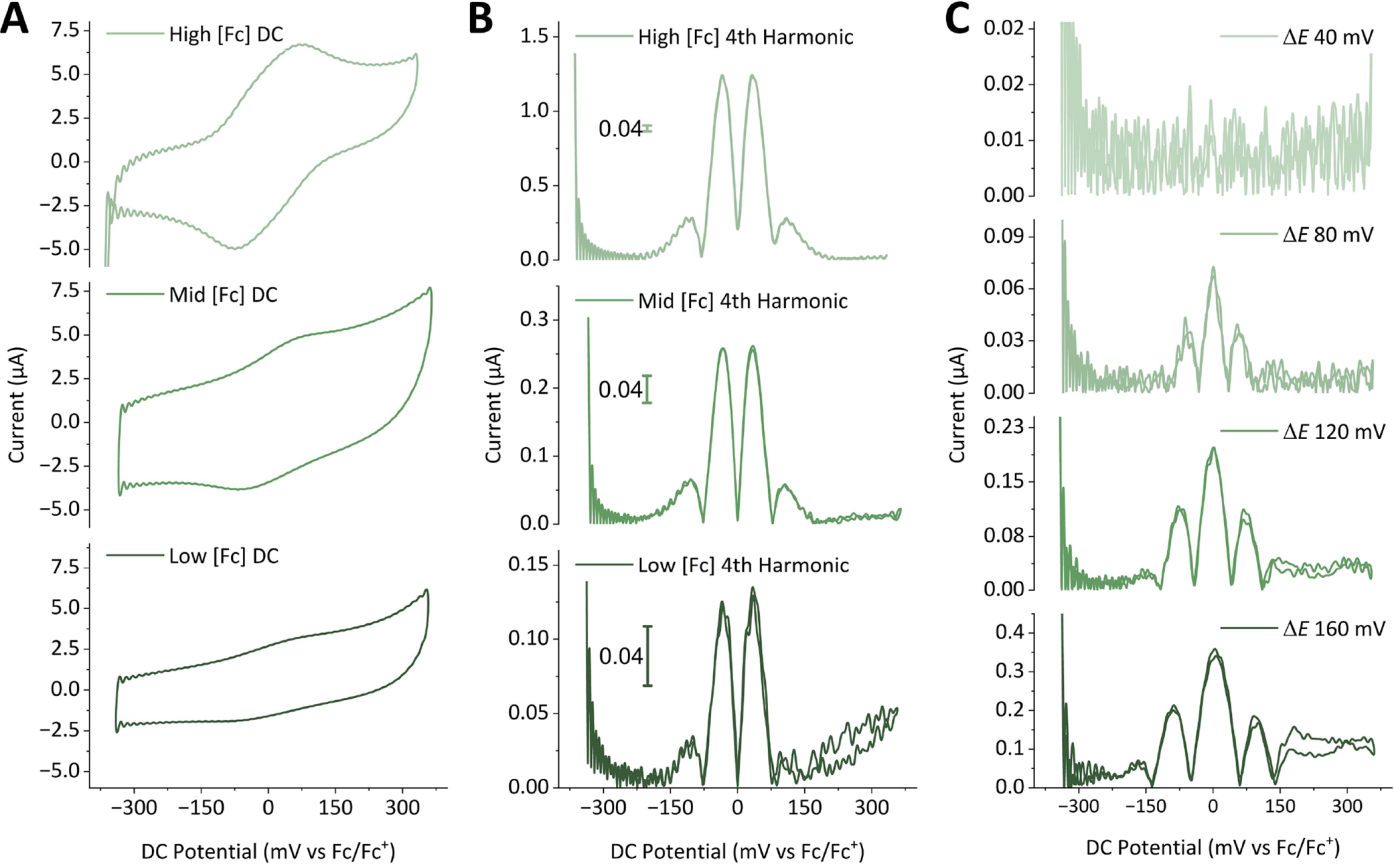
Experimental data showcasing the sensitivity of FTacV to Faradaic processes in low concentrations of the analyte and the effect of amplitude manipulation on harmonic plots. The data sets were collected on a reversible Fc^0/+^ process in MeCN containing 0.25 M [NBu_4_][PF_6_] as the supporting electrolyte, using the “Home-Build” instrument; ω = 72.05 Hz, Δ*E* = either 80 mV (A and B), or 40, 80, 120 or 160 mV (C), *v* = 104.31 mV s^–1^, DC potential range = −0.35–0.35 V vs Fc/Fc^+^. The ferrocene concentration was either 0.1 mM (“high [Fc]”), 0.01 mM (“mid [Fc]”), or 0.005 mM (“low [Fc]”). (A) Aperiodic DC components of the FTacV experiment as well as (B) envelope plots of the 4th harmonic at varying concentrations; obtained from FFT processing of the current–time output data, and subsequent iFFT of extracted harmonics. (C) 4th harmonic FTacV data collected for a 0.005 mM ferrocene solution, where Δ*E* was varied as indicated; harmonics were obtained in the same manner as those in (B).

Inspired by the comprehensive guide to cyclic voltammetry written by Elgrishi et al.,^1^ the data for the reversible Fc^0/+^ process provided in Figures 3 and 4 were collected using 0.1 mM ferrocene as an optimal concentration for the dcV experiments, providing a good Faradaic to background current ratio. One of the core advantages of FTacV is an improved ability to resolve Faradaic and capacitive contributions relative to dcV via the Fourier transform. The physical process by which the double-layer capacitance effect produces current is complex and challenging to model accurately. Lack of an accurate model makes resolving contributions from Faradaic and capacitive processes difficult, and consequently, many methods of electrochemical analysis assume a purely Faradaic current. Consequently, when attempting to extract quantitative information from electrochemical experiments, care is often taken to exclude or reduce such contributions. To demonstrate the enhanced sensitivity provided by FTacV,^32^ we have repeated the experiment from Figure 4, at lower concentrations of Fc, while leaving the remaining parameters constant. The enhanced sensitivity is shown in Figure 7A,B; as the ferrocene concentration is decreased from 0.1 mM (high [Fc]) to 0.01 mM (mid [Fc]) to 0.005 mM (low [Fc]), the current corresponding to the Faradaic response diminishes significantly across all current outputs (exemplified by inspection of the *y*-axis of the fourth harmonic data set, Figure 7B). However, because the fourth harmonic does not contain any substantive non-Faradaic contributions (it is effectively baseline-free), the clarity of the current signal from the ferrocene electron transfer process is overwhelmingly greater than in the aperiodic DC component from the same experiment (Figure 7A versus Figure 7B). Crucially, the signal resolution from high harmonic components of low Fc concentration FTacV experiments remains large enough to permit simple and accurate readout of the midpoint potential even under conditions where this would be impossible using dcV (the full data profile is available in Figures S6 and S7).

The sensitivity of an FTacV experiment can be further increased by tuning the amplitude parameter, Δ*E*, as demonstrated in Figure 7C. Comparison of the fifth harmonic from 0.005 mM ferrocene measurements across the amplitude range from 40 to 160 mV demonstrates that increasing the amplitude of the sine wave increases the magnitude of the Faradaic response. This is easily understood from the mathematical description of the Faradaic nonlinearity discussed above. However, it should be noted that increasing the amplitude also further exposes the nonlinearity of the capacitive response, as evidenced by the large nonferrocene signal in the Δ*E* = 160 mV experiment in Figure 7C (regions more positive than a DC potential of approximately 130 mV vs Fc/Fc^+^). Increasing the amplitude additionally increases the “width” of the harmonic current versus the DC potential; the effect is most apparent in Figure 7C for the 160 mV amplitude. Faradaic current is observed over a window of potential values, and at higher amplitudes, the FTacV input overlaps with this window for a greater portion of the DC ramp, an effect that is independent of capacitance. Consequently, harmonic current signals are observed at more values of the DC potential than in a lower amplitude experiment. The appropriate experimental value for the amplitude is a compromise between maximizing the Faradaic signal while making sure it is not overwhelmed by capacitive processes. In addition, larger amplitudes will increase the potential window of the experiment, and as such, care must be taken not to include potentials at which solvent breakdown is initiated. Thus, optimization of FTacV experimental parameters still requires analyte-free control experiments to be carried out as one would when optimizing any electrochemical methodology. Finally, in terms of the scan rate of an FTacV experiment, the value of this input parameter should be chosen after the desired frequency has been selected. This is primarily to avoid the overlap of the DC and AC time scales so that in the Fourier spectrum the aperiodic component is fully resolved from that of the AC fundamental harmonic. A rule of thumb is that Δ*E*ω ≫ *v*,^41^ with more detailed simulation studies suggesting a lower bound of 512 sinusoidal oscillations per DC sweep to resolve the second harmonic under small amplitude conditions.^42^ In this work, we have used approximately 500 oscillations per sweep.

**Figure 8:**
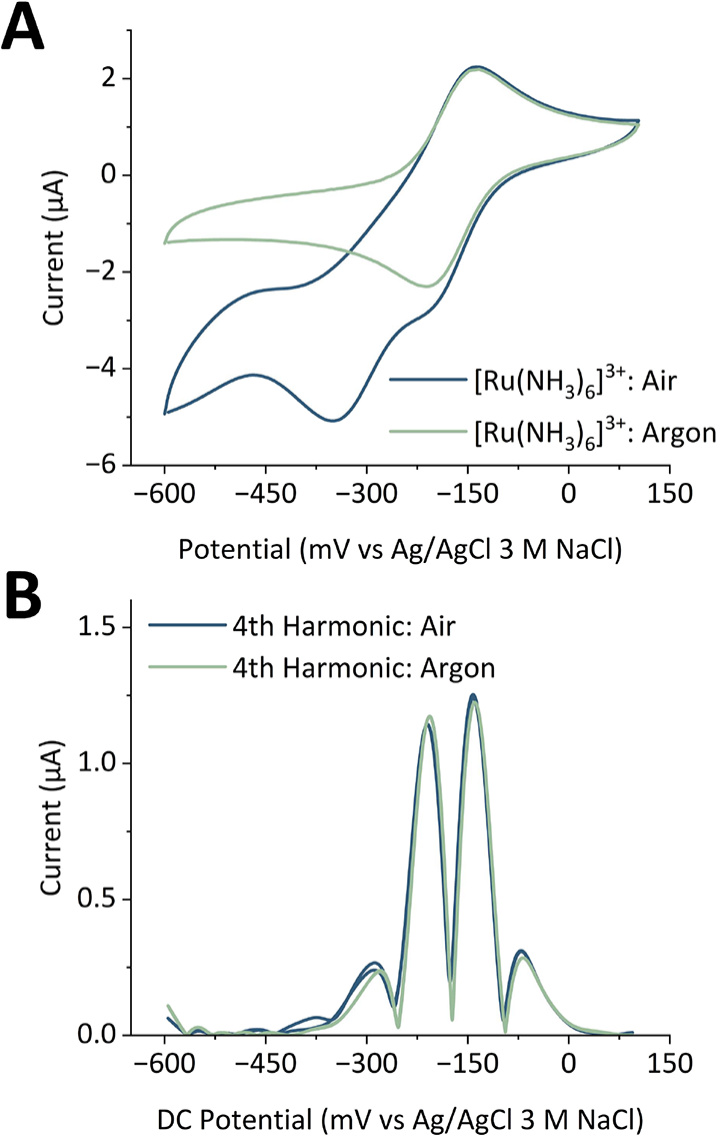
(A) dcV data for a reversible [Ru(NH_3_)_6_]^3+/2+^ process in an aqueous solution containing 0.5 M KCl as the supporting electrolyte, where [M] = 0.20 mM, collected on the “Ivium” potentiostat; *v* = 52.15 mV s^–1^, linear potential range = −0.6–0.1 V vs Ag/AgCl 3 M NaCl; the data was collected in the presence of air (blue) and under an argon atmosphere (green). (B) Corresponding FTacV data on the same solution collected on the “Home-Build” potentiostat; ω = 9.54 Hz, Δ*E* = 80 mV, *v* = 52.15 mV s^–1^, DC potential range = −0.6–0.1 V vs Ag/AgCl 3 M NaCl. The envelope of the 4th harmonic is shown, obtained from the FFT processing of the current–time output data and subsequent iFFT of the extracted 4th harmonic.

In Figure 3, we demonstrate that FFT and subsequent iFFT processing of FTacV data enable the effective isolation of the Faradaic current from non-Faradaic current contributions. In Figure 8, we illustrate another application of this filtering approach: the ability to separate slow and rapid electron-transfer processes that are overlapping in dcV. Inspired by a previously published work,^8^ the data in Figure 8 was obtained from a [Ru(NH_3_)_6_]^3+/2+^ redox process containing 0.20 mM of the analyte in an aqueous solution containing 0.5 M KCl as the supporting electrolyte under an atmosphere of air and an atmosphere of argon. While the former experimental setup is trivial to achieve (i.e., the electrochemical cell could be a simple beaker open to the lab atmosphere), the latter requires a sealed system, access to compressed gas and controlled monitoring of the gas flow. As shown in Figure 8A, under an atmosphere of air, the dcV experiment exhibits a complex, “non-duck”-shaped current response that results from two contributing electron-transfer processes occurring over the same potential window. The first electron transfer is the reversible one-electron [Ru(NH_3_)_6_]^3+/2+^ process, which overlaps with the irreversible reduction of oxygen at the glassy carbon working electrode. This overlap precludes accurate determination of the redox chemistry of [Ru(NH_3_)_6_]^3–^ using dcV analysis of an experiment carried out in air.

The presence of a proton source such as water makes oxygen reduction a relatively prominent electrocatalytic reduction process on carbon, gold, and platinum working electrodes. Therefore, to use dcV to study the redox chemistry of analytes such as [Ru(NH_3_)_6_]^3+^ in aqueous electrolyte media, an argon or nitrogen atmosphere must be used to displace oxygen, as shown in Figure 8A. In stark contrast, a comparison of the harmonics of FTacV [Ru(NH_3_)_6_]^3+^ experiments under either an air or an argon gas atmosphere demonstrates very little change with the gas atmosphere (Figure 8B, the fourth harmonic is shown as an example). The [Ru(NH_3_)_6_]^3+^ electron transfer is readily separable from the oxygen reduction reaction when using FTacV as a result of the differing kinetic properties of the two processes. The current contribution from the latter irreversible process is minimal in the higher harmonics at a frequency of 9.54 Hz where there are minimal contributions from the kinetically slow oxygen reduction process.

## Conclusion

Voltammetry is a remarkably powerful technique for simultaneously investigating both the thermodynamics (potential driving force) and kinetics (electric current) of electron transfer processes, a class of chemical reactions which underpin energy technology, electrosynthetic methodologies and the mechanisms of life. A fundamental limitation to dcV, the simplest voltammetric technique, is that under certain experimental conditions, the non-Faradaic “background” current contribution overwhelms the Faradaic current. This is a substantive issue in our own work on the film voltammetry of redox-active proteins and enzymes and can also be a major issue for chemists aiming to investigate the redox behavior of small amounts of novel compounds. We wish to showcase how it is possible to utilize the FTacV technique to overcome difficulties with some of the sensitivity limitations associated with dcV experiments in studies of fast processes. However, it must be noted that studies of highly irreversible processes would preferably be undertaken by dcV methods.

As noted in the introduction, the form of FTacV described in this paper employs a sine wave superimposed onto a DC ramp. However, square-wave voltammetry (SWV) experiments in which a combined square wave and linear-staircase potential are applied to a working electrode can also be subjected to FTacV protocols to give a direct distribution of harmonic content.^30^ However, most versions of the square-wave method simply sample the current at the end of each square wave step, rather than for the whole duration of the square wave as required in its FTacV approach. This simplified SWV protocol makes it relatively easy to perform accurate measurements using almost any commercial instrument, whereas to date FTacV has not been commonly integrated into method options available in off-the-shelf instrumentation. Correspondingly, while there is a wealth of excellent review papers,^43−45^ books,^46,47^ and technical reports^48,49^ which introduce a newcomer to the details of how and why one may make a SWV measurement using a two-point form of data measurement, there has been no comprehensive “introductory guide” to conducting FTacV experiments with commercial instruments. Although there are many instances where using SWV over dcV may provide an experimentalist with enough of an increase in experimental sensitivity, the enhanced kinetic resolution provided by FTacV means that this is an invaluable extra voltammetric technique which we hope will become more accessible based on this study. We hope that the fundamental information provided, with easy to conduct check-cell and ferrocene experiments and illustrations of the effect of low sampling frequency/data buffer sizes, will facilitate more experimentalists in conducting FTacV experiments accurately utilizing modern potentiostats. Computational software and hardware advances also facilitate the ever-simpler processing of FTacV data, and we provide a comprehensive SI and PuRe data repository (**DOI:** 10.15124/194bb079–32f5–420b-a2e0-e3d53b3cdfbd) in accompaniment to this paper. We intend that newcomers to the technique should not have to start from scratch in figuring out how to easily perform FFT processing to generate a Fourier spectrum, band selection and iFFT to isolate the separate harmonic signals, instead, they can refer to the supplied processing code. We note that this “Practical Guide” does not comprehensively explore all possible instrumental limitations which may complicate the adaptation of a commercial instrument for FTacV. Therefore, we refer interested readers to the comprehensive introduction to the technique published in 2005 and note that this provides many important details on instrument design.^9^

In terms of the exemplar solution electrochemistry experiments shown here, we bring together a collection of measurements inspired by many years of method development. As explained in simplistic terms in this paper, the enhanced sensitivity and selectivity of FTacV measurements arise from the fact that the FFT and iFFT data analysis protocol separates out current contributions from processes with different time constants. Thus, selecting the ideal frequency and amplitude of an FTacV experiment is akin to tuning a radio; you can “listen” to the current-generating processes of interest by “dialling in” the right sine wave parameters. We and others have found FTacV to be a particularly powerful method in the bioelectrochemical characterization of redox-active metalloenzymes and proteins.^20,21,23,34−36,50^ Other examples illustrating the power of this technique can be found in studies of heterogeneous and homogeneous redox catalysis, with high-impact work on fuel cells^25^ and carbon dioxide reduction catalysis.^37,51^

As is described in detail in previous work,^6,10,17,18,52−54^ it is possible to analyze data from FTacV experiments using simulation approaches in order to determine precise mechanistic details (number of electrons, precise midpoint potentials, electron transfer kinetic regimes, etc.). This represents one of the major advantages of the technique in comparison to the widely used forms of SWV, but it is simply beyond the scope of this work to also describe simulation methodologies, especially given the range of nonidealities displayed by the commercial instruments. Indeed, the first step of such simulation experiments would be to independently validate the potential–time input from the instruments versus the recorded potential–time input. Instead, we refer interested readers to our previous extensive work on the large amplitude form of FTacV and note that under the conditions described herein, the Fc^0/+^ and [Ru(NH_3_)_6_]^3+/2+^ systems will be reversible or at equilibrium at low frequencies. Thus, they can be use as reference systems to test instrumentation idealities in organic solvents or aqueous media, respectively.

## Data Availability

The data underlying this study are openly available in PuRE at DOI: 10.15124/194bb079–32f5–420b-a2e0-e3d53b3cdfbd.

## Supporting Information Available

The Supporting Information (SI) is available free of charge. The SI contains (**1**) details of the check-cells; (**2**) annotated Python code for the processing and plotting of the data; (**3**) Nyquist frequency optimization; (**4**) additional harmonic plots for the experiments shown in Figure 7 (PDF)
