# Supplementary material for: Practical Guide to Large Amplitude Fourier-Transformed Alternating Current Voltammetry—What, How, and Why
Source: ACS Meas Sci Au. 2024 May 7;4(4):418–31. doi: 10.1021/acsmeasuresciau.4c00008 (PMC11342453; doi:10.1021/acsmeasuresciau.4c00008)
Supplement: Supplementary file 1 — tg4c00008_si_001.pdf [file tg4c00008_si_001.pdf]

Supporting Information (SI)

A Practical Guide to Large Amplitude Fourier-Transformed Alternating Current Voltammetry – What, How and Why

Natalia G. Baranska[a], Bryn Jones[b], Mark R. Dowsett[c], Chris Rhodes[a], Darrell M. Elton[d], Jie Zhang[e], Alan M. Bond[e], David Gavaghan[f], Henry O. Lloyd-Laney[a*], Alison Parkin[a*]

[a] Department of Chemistry, University of York, Heslington, York YO10 5DD, United Kingdom

[b] SciMed, Unit B4, The Embankment Business Park, Vale Road, Heaton Mersey SK4 3GN, United Kingdom

[c] Alvatek Ltd, Unit 11 Westwood Court, Brunel Road, Southampton, SO40 3WX, United Kingdom

[d] School of Engineering and Mathematical Sciences, La Trobe University, Bundoora, Victoria 3086, Australia

[e] School of Chemistry and the ARC Centre of Excellence for Electromaterials Science, Monash University, Clayton, Victoria 3800, Australia

[f] Department of Computer Science, University of Oxford, Wolfson Building, Parks Road, Oxford OX1 3QD, United Kingdom

Check-Cell Design………………………………………………………………………… S2

FTacV Scripting and Data Plotting Methods…………………………………………… S3

(i) Generating a Potential Input…………………………………………………… S3

(ii) Check-Cell Validations………………………………………………………… S5

(iii) Plotting Time-Potential-Current……………………………………………….. S7

(iv) Applying Windowing Functions………………………………………………... S7

(v) Plotting the Fourier Spectrum…………………………………………………. S8

(vi) Applying Filters to Extract Fourier-Domain Harmonics……………………... S9

(vii) Plotting Time-Domain Harmonics……………………………………………... S10

Optimising the Nyquist Frequency on a FTacV Experiment………………………….. S10

FTacV Measurements at Different Ferrocene Concentrations………………………. S12

CHECK-CELL DESIGN

In the main paper, we describe simple confirmation experiments performed using a check-cell that can be configured to give either a series "RC_{ideal}" circuit or a series "$RC_{non\text{-}ideal}$" circuit, as illustrated in **Figure S1**. In both cases, the resistor (R1 in **Figure S1 A**) is the same "10 Ω" component (measured as 10.5 Ω using a multimeter) (VISHAY, AC04AT0001009JAC00, through hole resistor, 10 Ω, AC-AT, 4 W, ± 5%, axial leaded) while the "ideal" capacitor (C1 in **Figure S1 A**) is a metallized polypropylene "10 µF" component (measured as 10.1 µF using a multimeter) (EPCOS, B32774H4106K000, power film capacitor, metallised PP, radial box - 2 pin, 10 µF, ± 10%, DC link, through hole), while the "non-ideal" capacitor (C2 in **Figure S1 A**) is a ceramic "10 µF" component (measured as 9 µF using a multimeter) (TDK, FG28X5R1E106MRT06, multilayer ceramic capacitor, 10 µF, 25 V, ± 20%, PC pin, X5R, 5 mm).

(A)

(B)

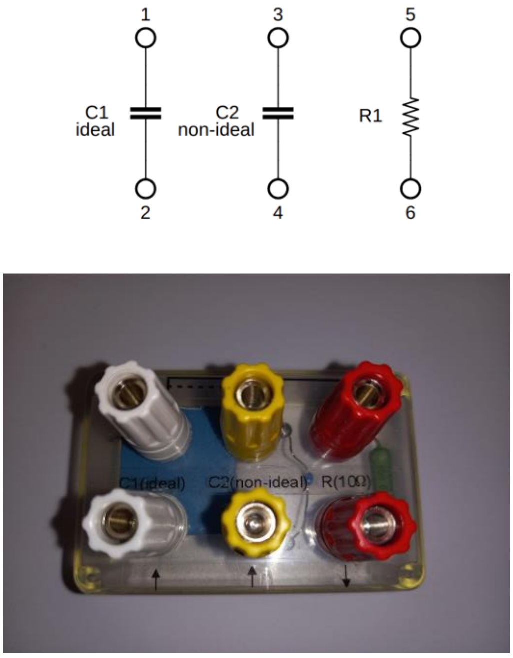

Figure S1. (**A**) Circuit diagram and (**B**) photograph of the check-cell used in this study. Cables are used to connect the instruments tested to the check-cell in either a series RC_{ideal} circuit or a series $RC_{non\text{-}ideal}$ circuit, as described in the main paper.

FTacV SCRIPTING AND DATA PLOTTING METHODS

In this section, the methods used to proceed through the workflow of an FTacV experiment are described; the overall process is summarised in **Figure S2**.

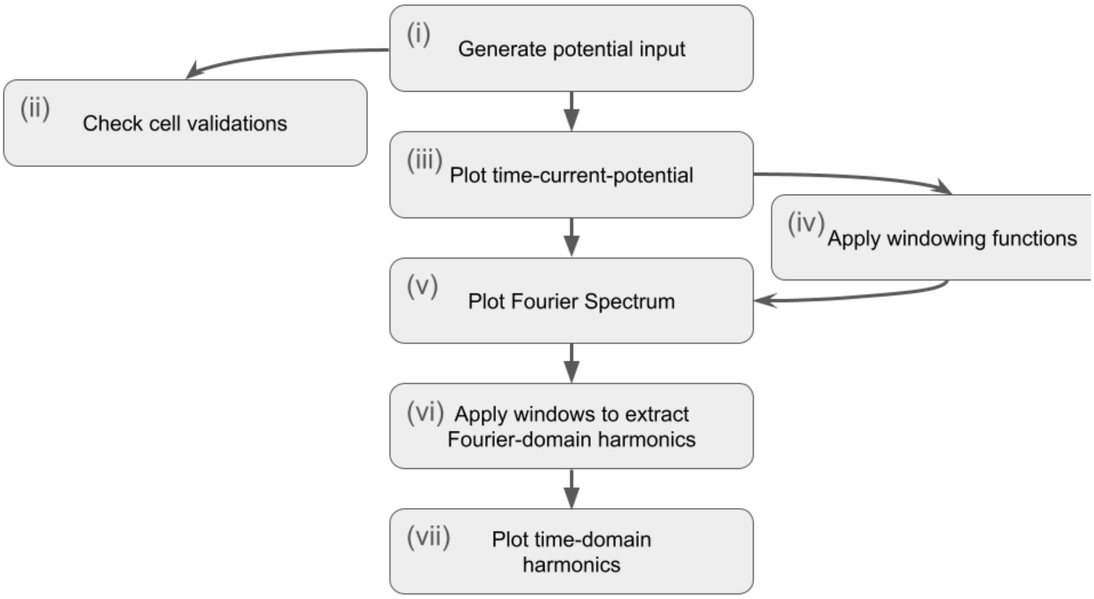

Figure S2. A schematic of the overall workflow needed to conduct an FTacV experiment and visualise the data in the manner shown in the main paper.

SCRIPTING & DATA PLOTTING: (i) GENERATING A POTENTIAL INPUT

The method by which the potential-time input is defined will depend on the potentiostat being used. Many manufacturers provide the option to apply a user-defined sequence of time-potential values, and as such we provide the below python code. The input frequency is often selected on the basis of the expected value of the electron-transfer rate constant, as discussed in the main paper. The scan rate should be slower than the time-constant of the frequency for two reasons. If the scan rate is too fast then the potential input doesn't spend enough time in the Faradaic window, reducing the amount of information obtainable from harmonic analysis. In addition, the faster the scan rate, the broader the width of the 0 Hz harmonic (referred to as aperiodic DC component in the main text) in the frequency spectrum; at inappropriate combinations of the scan rate and the frequency the 0 Hz harmonic can overlap with the fundamental harmonic, making analysis challenging.

```
import numpy as np

#########################USERVALUES################################

min_interval=1/5000 #Delta T in seconds. The minimum value of this will be defined by the instrument

desired_Hz=10 #Input frequency in Hz

desired_scan_rate=22.5e-3 # Scan rate in V/s

E_reverse=0.5 # Switching potential in V

E_start=-0.2 # Start potential in V

phase=0 # Phase of the input sinusoid

strictly_periodic=True # For Fourier analysis, you may want to enforce

periodicity of your sine wave.

Ac_amplitude=0.15 # Amplitude of the sine wave in volts. Check with the manufacturer as to how they define this value

######################################################################


phase_rad=phase*(np.pi/180)

distance=(E_reverse-E_start)

DC_range=distance*2

end_time=DC_range/desired_scan_rate

if strictly_periodic==True:

   end_time=end_time-(end_time%(1/desired_Hz))

   actual_scan_rate=distance/(end_time/2)

else:

   actual_scan_rate=desired_scan_rate

num_points=int(end_time//min_interval)+1


t=np.linspace(0, end_time,num_points)

switch_time=t[len(t)//2]

E=E_reverse-(actual_scan_rate*np.abs(t-
switch_time))+Ac_amplitude*np.sin((desired_Hz*2*np.pi*t)+phase_rad)
```

```
with open("Potential_values.txt", "w") as f:

   np.savetxt( f,np.column_stack((t, E)))
```

SCRIPTING & DATA PLOTTING: (ii) CHECK-CELL VALIDATIONS

In the main paper we show data in **Figure 5** that compares the consistency with which a sinusoidal oscillation is achieved throughout an FTacV experiment by the three different instruments tested. We extract the phase of each sinusoid in the applied potential-time dataset using a lock-in amplifier method to determine the phase of each sinusoid in a potential input and the corresponding current output, as detailed below.

```
freqs=np.fft.fftfreq(len(voltage), time[1]-time[0])

potential_Y=np.fft.fft(voltage)

current_Y=np.fft.fft(current)

fundamental_hz=freqs[np.where(Y==max(Y[np.where(freqs>10)]))][0]#obtain
ing fundamental frequency

potential_Y[np.where((freqs<0.25*fundamental_hz) & (freqs>-
0.25*fundamental_hz))]=0

ac_component=np.real(np.fft.ifft(potential_Y))#Removing the DC
component

#If the signal is aperiodic you will get "ringing" elements at the
start

#and end of the timeseries. For this analysis, it is recommended you

#truncate the signal accordingly

num_periods=int(np.floor(time[-1]*fundamental_hz))

periods=list(range(1, num_periods))

phases=np.zeros((2, num_periods-1))

for i in range(0, num_periods-1):

   idx=np.where((time>(i/fundamental_hz))&

      (time<((i+1)/fundamental_hz)))

   s=np.sin(2*np.pi*fundamental_hz*time[idx])

   c=np.cos(2*np.pi*fundamental_hz*time[idx])
```

```
    sines=[current[idx], ac_component[idx]]

    #To verify this method works uncomment these two lines

    #check_phase=70

    #sines[1]=np.sin(2*np.pi*fundamental_hz*time[idx]

       +(check_phase*(np.pi/180)))


    for m in range(0, len(sines)):


        sinusoid=sines[m]

        xs=sinusoid*s

        xc=sinusoid*c

        a=2*np.mean(xs)

        b=2*np.mean(xc)

        mag=np.hypot(b,a)

        rad=np.arctan2(b,a)

        deg=rad*180/np.pi

        phases[m][i]=deg
```

The reason(s) that the Ivium and Gamry instruments tested generate a sinusoidal-portion of the FTacV potential-time input which has an apparently changing phase as a function of time (**Figure 5** in the main paper) is still unclear. We are investigating compensatory feedback mechanisms within the instruments. It is notable that irregularities in the apparent phase of the commercial potentiostats vary as a function of the input FTacV experiment parameters, as shown in **Figure S3**.

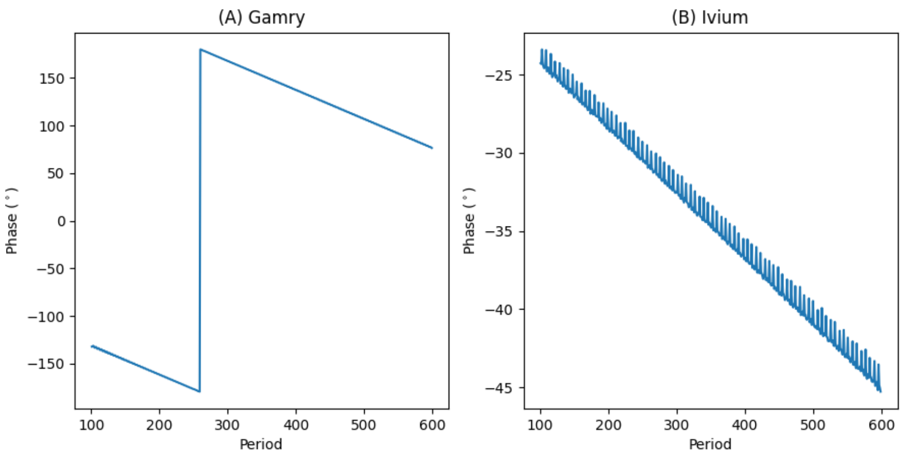

Figure S3. Plots showing the apparent variation in the input potential phase as a function of time. Experiments were conducted on the ideal check cell circuit. The FTacV parameters used are different for the two instruments, since they were selected to elicit very large apparent phase shifting. (**A**) Data obtained from the Gamry instrument using a voltage window of -0.5 – 0.5 V, an input frequency of 120 Hz and an amplitude of 100 mV. (**B**) Data obtained from the Ivium instrument using a voltage window of 0 – 1 V, an input frequency of 72 Hz and an amplitude of 100 mV.

SCRIPTING & DATA PLOTTING: (iii) PLOTTING TIME-POTENTIAL-CURRENT

This is trivial to do in any plotting software, but it is the opportunity for some important checks:

- That the potential has been applied properly, for the correct period of time, over the correct potential range and with the correct amplitude.
- That there is not significant noise in the applied potential.
- Usually (unless operating at very low concentrations/high resistances/high background currents) it will be possible to observe a Faradaic peak at the midpoint potential in the total experimental current.

SCRIPTING & DATA PLOTTING: (iv) APPLYING WINDOWING FUNCTIONS

When plotting harmonics, “ringing” artefacts of large amplitude at the start and end of the time series can be observed, as shown in **Figure S4**. These artefacts can be large enough to obscure the actual harmonic signal, and arise from aperiodicity in the current signal. Such ringing effects can be removed by applying the Hanning window, as shown in Figure S4, although care must be taken; the window suppresses the magnitude of the current linearly, such that the beginning and end of the signal are set

to zero. For example, for the 0th harmonic component, the use of a Hanning filter can completely suppress the Faradaic peak. In python the Hanning window can be trivially applied using numpy:

```
import numpy as np

hann_window=np.hanning(len(total_current))

windowed_current=np.multiply(total_current, hann_window)
```

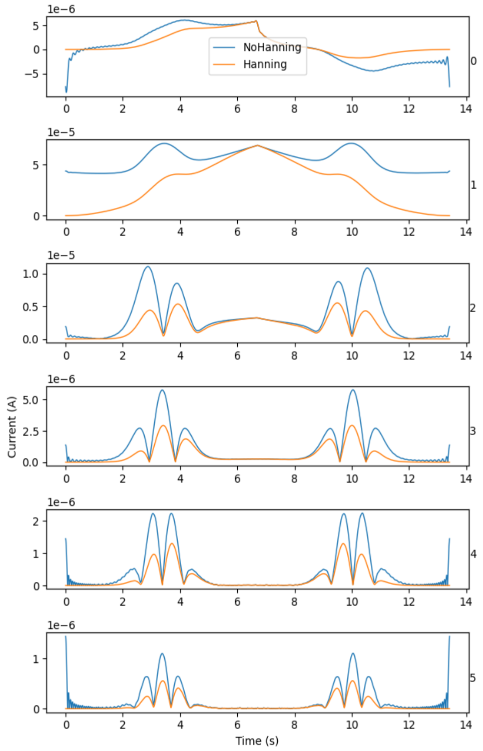

Figure S4. Effect of applying a Hanning window (orange line) versus no Hanning window (blue line) for harmonics 0-5 (top to bottom). The Hanning window is used to reduce the size of the ringing artefacts at the beginning and end of the harmonics, by suppressing the start and end of the total current to zero. However, it also has the effect of suppressing harmonic magnitudes, and introducing asymmetry into the harmonic lobes. The harmonics are obtained from the data plotted in **Figure 1** in the main paper.

SCRIPTING & DATA PLOTTING: (v) PLOTTING THE FOURIER SPECTRUM

Investigating the Fourier spectrum of the total current is another useful check of the validity of the FTacV experiment. Visually inspecting the Fourier spectrum allows an experimentalist to verify the presence of harmonics, determine the true input frequency applied by the potentiostat (by checking the frequency at which the fundamental harmonic occurs in the Fourier spectrum), and assess at which harmonic number the

Fourier spectrum peak can be meaningfully separated from the baseline noise level in the spectrum. To plot in python:

```
import numpy as np

import matplotlib.pyplot as plt

FT=np.fft.fft(windowed_current)

timestep=time[1]-time[0]

frequencies=np.fft.fftfreq(len(windowed_current), timestep)

plt.plot(np.fft.fftshift(frequencies),
np.fft.fftshift(windowed_current))

plt.show()
```

SCRIPTING & DATA PLOTTING: (vi) APPLYING FILTERS TO EXTRACT FOURIER-DOMAIN HARMONICS

In FTacV analysis, we wish to ultimately obtain a signal that is the same length as the number of timesteps/applied potential values. To do so, we create an m*n array of complex zeros, where m is the number of harmonics we want to plot, and n is the length of the input current. We then select the appropriate “box” around each desired harmonic (at this point you may wish to apply more complex filters) and assign this to the appropriate position in the m*n array.

```
import numpy as np

import matplotlib.pyplot as plt

desired_harmonics= np.arange(0, 8, 1)#Plotting harmonics 0 to 7

num_desired_harmonics=len(desired_harmonics)

fft_harmonics=np.zeros((num_desired_harmonics,frequencies),dtype="compl
ex") # The fourier spectrum is made up of complex numbers

box_width=0.5*desired_Hz # The region of the Fourier spectrum that we
will extract around each harmonic as a fraction of the input frequency

envelope=True

for i in range(0, num_desired_harmonics):

   harmonic_i=i*desired_Hz
```

```
    if envelope==True:
        signs=[1]
    else:
        signs=[-1,1]
    for sign in signs:
        box_lower_bound=sign*harmonic_i-box_width
        box_upper_bound=sign*harmonic_i+box_width
        box_region=np.where((one_tail_frequency>box_lower_bound) &
             (one_tail_frequency<box_upper_bound))
        fft_harmonics[i,box_region]+=FT[box_region]#Only the frequency
regions that are defined by the box are copied over - everything else
stays as complex 0
```

SCRIPTING & DATA PLOTTING: (vii) PLOTTING TIME-DOMAIN HARMONCIS

The script we use to generate the individual time-domain harmonics is shown below.

```
for i in range(0, num_desired_harmonics):
    if envelope==True:
        plot_harmonic= 2*np.abs(np.fft.ifft(fft_harmonics[i,:]))
    else:
         plot_harmonic= np.real(np.fft.ifft(fft_harmonics[i,:]))
    plt.plot(time,plot_harmonics) # We can now plot the time-domain
representation (obtained using ifft) using an appropriate function
    plt.show()
```

OPTIMISING THE NYQUIST FREQUENCY OF A FTacV EXPERIMENT

As detailed in the main paper, both the frequency of the maximum recoverable harmonic and the signal-to-noise resolution of an FTacV experiment is limited by the Nyquist frequency, which is half the sampling frequency. Amongst many other limitations which may impact an FTacV experiment, an instrument will have a maximum sampling frequency, and a maximum number of data points which it can

store in memory. The total number of datapoints required for an FTACV experiment is given by the sampling frequency multiplied by the experiment time; in FTacV the experiment time is determined by the time taken to complete the DC potential cycle. To illustrate this and assist researchers in considering the maximum harmonic they can theoretically access according to the Nyquist frequency, **Figure S5** shows the number of points required to access the 30th harmonic as a function of input frequency and scan rate for a theoretical experiment measured over a linear potential window of 1 V. The number of points required for a particular set of parameters can be easily calculated using the code for potential generation found above. As noted above, we

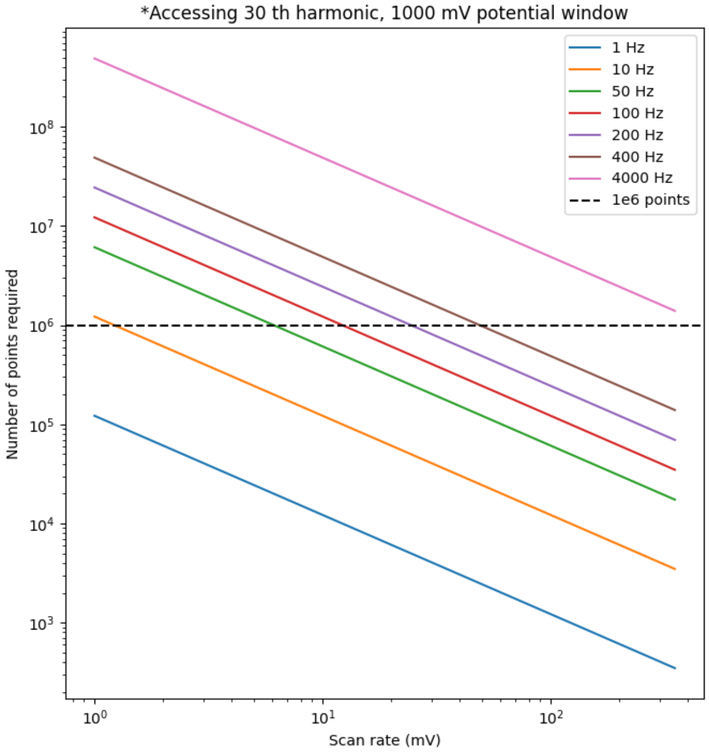

Figure S5. Number of points required to access the 30th harmonic as a function of input frequency and scan rate for a theoretical experiment measured over a linear voltage window of 1 V. The maximum number of points collectable by the Gamry potentiostat (1 million) is shown as a horizontal line.

emphasise that caution must be taken if using a fast a scan rate in FTacV; it is important that the Fourier spectrum is always carefully analysed to ensure that a combination of fast linear scan rate and high frequency does not cause an overlap in current contributions from the 0th/aperiodic DC component and the 1st/fundamental harmonic.

FTacV MEASUREMENTS AT DIFFERENT FERROCENE CONCENTRATIONS

While **Figure 7** of the main paper only shows the aperiodic DC component and 4th harmonic of FTacV experiments conducted at three different concentrations of ferrocene, **Figure S6** displays a more complete data profile to assist researchers in understanding how to use FTacV to accurately determine midpoint potentials using FTacV in concert with low analyte concentrations. For completeness, **Figure S7** is included to show the effect of a changing amplitude on harmonics 0-7.

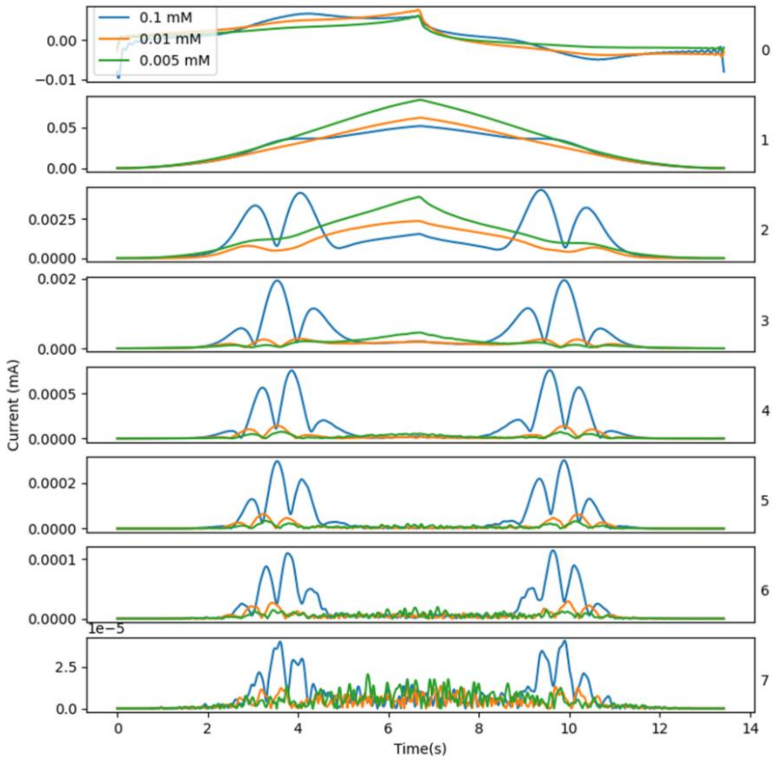

Figure S6. Harmonics 0-7 as a companion to **Figure 7B** showing the impact of reducing ferrocene concentrations, as indicated. All experiments were conducted at 72 Hz, with a scan rate of 104.31 mV s^{-1}, an amplitude of 80 mV and over a potential window of 0.35 – 1.05 V vs Ref on the Home-built instrument; Hanning window was applied.

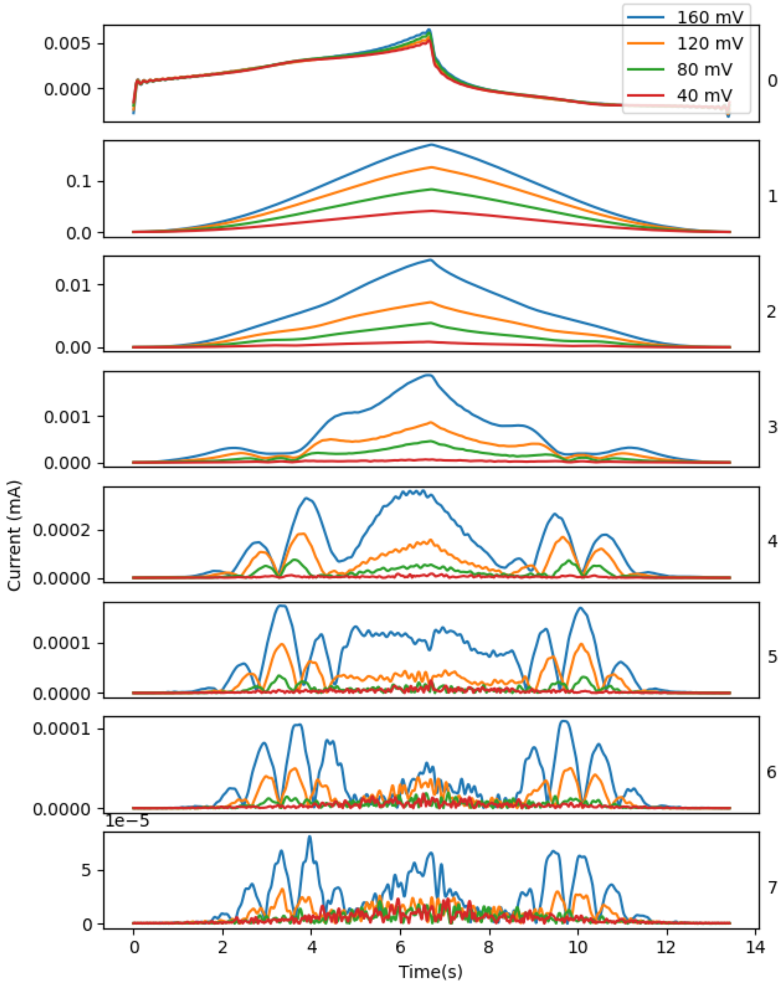

Figure S7. Harmonics 0-7 as a companion to **Figure 7C** showing the impact of increasing the amplitude of the sine in the potential input, as indicated. All experiments were conducted at 72 Hz, with a scan rate of 104.31 mV s^{-1} and over a potential window of 0.35 – 1.05 V vs Ref on the Home-built instrument at a ferrocene concentration of 0.005 mM; Hanning window was applied.
